# Prevalence and Determinants of Cervicovaginal, Oral, and Anal Human Papillomavirus Infection in a Population of Transgender and Gender Diverse People Assigned Female at Birth

**DOI:** 10.1089/lgbt.2023.0335

**Published:** 2024-09-05

**Authors:** Ryan D. McIntosh, Emily C. Andrus, Heather M. Walline, Claire B. Sandler, Christine M. Goudsmit, Molly B. Moravek, Daphna Stroumsa, Shanna K. Kattari, Andrew F. Brouwer

**Affiliations:** ^1^ Department of Epidemiology, University of Michigan, Ann Arbor, Michigan, USA.; ^2^ Department of Otolaryngology, University of Michigan, Ann Arbor, Michigan, USA.; ^3^ Reproductive Endocrinology Clinic, Center for Reproductive Medicine, University of Michigan, Ann Arbor, Michigan, USA.; ^4^ School of Social Work, University of Michigan, Ann Arbor, Michigan, USA.; ^5^ Department of Women's and Gender Studies, University of Michigan, Ann Arbor, Michigan, USA.

**Keywords:** cancer screening, cervicovaginal, human papillomavirus (HPV), self-sampling, transgender and gender diverse (TGD)

## Abstract

**Purpose:**

The human papillomavirus (HPV) causes cervicovaginal, oral, and anogenital cancer, and cervical cancer screening options include HPV testing of a clinician-collected sample. Transgender and gender diverse (TGD) people assigned female at birth (AFAB) face many barriers to preventive care, including cancer screening. Self-sampling options may increase access and participation in HPV testing and cancer screening. This study estimated the prevalence of HPV in self-collected cervicovaginal, oral, and anal samples from Midwestern TGD individuals AFAB.

**Methods:**

We recruited TGD individuals AFAB for an observational study, mailing them materials to self-collect cervicovaginal, oral, and anal samples at home. We tested samples for high-risk (HR; 16, 18, 31, 33, 35, 39, 45, 51, 52, 56, 58, 59) and other HPV genotypes (6, 11, 66, 68, 73, 90) using a polymerase chain reaction mass array test. Prevalence ratios for HPV infection at each site as a function of participant characteristics were estimated in log-binomial models.

**Results:**

Out of 137 consenting participants, 102 completed sample collection. Among those with valid tests, 8.8% (HR = 6.6%; HPV 16/18 = 3.3%) were positive for oral HPV, 30.5% (HR = 26.8%; HPV 16/18 = 9.7%) for cervicovaginal HPV, and 39.6% (HR = 33.3%; HPV 16/18 = 8.3%) for anal HPV. A larger fraction of oral (71.4%) than anal infections (50.0%) were concordant with a cervicovaginal infection of the same type.

**Conclusions:**

We detected HR cervicovaginal, oral, and anal HPV in TGD people AFAB. It is essential that we reduce barriers to cancer screening for TGD populations, such as through the development of a clinically approved self-screening HPV test.

## Introduction

The human papillomavirus (HPV) causes more than 24,000 cancers each year in cisgender women and people assigned female at birth (AFAB), including 12,000 cervical cancers.^1^ The U.S. Preventive Services Task Force (USPSTF) recently revised cervical cancer screening guidelines to include the option of testing for high-risk (HR) HPV instead of, or in addition to, cytological (Pap) screening.^2^ Many physicians believe transmasculine people (AFAB individuals who identify as male or masculine^3^) and nonbinary and gender diverse people (those who identify between or beyond the male–female gender binary; note that transgender and gender diverse [TGD] may be overlapping identities) to be at a lower risk for cervical cancer than cisgender (i.e., nontransgender) women because of lower rates of penile–vaginal intercourse.^4^

However, prior studies have suggested that transmasculine populations have just as high or higher rates of sexually transmitted infections (STIs) compared to cisgender people and may be at equal or higher risk of HPV-associated cancers.^3,5^

Moreover, TGD people face discrimination and stigma in health care settings and daily life and consequently are less likely to seek reproductive and preventive health care services, including cervical cancer screening.^6–12^ Specific barriers to screening include fear of dysphoria and discomfort, fear of discrimination, and fear of providers lacking knowledge of care for TGD patients. Recent studies have proposed HPV self-sampling as an alternative to provider-based testing, circumventing some of these barriers.^13–16^ More work is needed to understand the risk of HPV-associated cancers in TGD populations^17^ as well as the impact and feasibility of HPV self-sampling as a cervical cancer screening tool. This work is especially crucial in the context of the updated USPSTF guidelines and the current politicization of TGD medical care.

High-risk (HR) HPV genotypes cause cancer (with the majority caused by genotypes 16 and 18), and some other types can cause genital warts.^1,18^ While HPV is best known as the etiological agent of nearly every cervical cancer, it also causes a large fraction of anal, genital, and oropharyngeal cancers.^1,18,19^ Oropharyngeal cancer is now the HPV-associated cancer with the highest incidence in the United States, with ∼19,000 of the 43,000 HPV-associated cancers diagnosed occurring at oropharyngeal sites (∼12,000 are cervical, 7000 are anal, and the rest are at genital sites).^20^ There is increasing recognition that we must consider HPV infection at multiple anatomical sites (cervicovaginal, oral, anal) to fully understand its epidemiology.^21–28^

We extend this perspective to HPV-associated cancer risk, screening, and prevention for TGD people AFAB. Our objective was to estimate the prevalence and determinants of cervicovaginal, oral, and anal HPV in a sample of Midwestern TGD people AFAB and to demonstrate the feasibility of at-home self-sampling for HPV testing in this population.

## Methods

### Study population

#### Eligibility criteria

To participate in the study, potential candidates had to meet all of the following criteria: (1) be between the ages of 21–65, (2) have been AFAB, (3) have a male, masculine, nonbinary, queer, or another gender diverse identity other than female or feminine, (4) not be pregnant, (5) not have had cervix removed, (6) not be menstruating on the day of the cervicovaginal swab self-collection, and (7) live in Michigan, Illinois, Indiana, Ohio, or Wisconsin. The geographic criterion was intended to distinguish this study from studies in coastal, urban populations. Individuals of any HPV vaccination status or prior HPV infection status were eligible.

#### Recruitment and enrollment

Recruitment methods included advertisements and listserv emails through Michigan Medicine's Comprehensive Gender Services Program, the University of Michigan Health Research posting site, social media posts, outreach to statewide lesbian, gay, bisexual, transgender, queer, and others groups and organizations, and word-of-mouth referrals. Interested participants reached out to study staff, who screened them for eligibility requirements. Potential participants were then invited to an individual virtual video to review the consent form and study details. All participants gave written, informed consent virtually, using SignNow.

#### Follow-up

Participants who did not complete the study were contacted up to three times before being marked as lost-to-follow-up.

### Surveys

Participants completed two surveys on an online platform (Qualtrics). The demographic and behavioral survey included questions on sociodemographic characteristics; health care utilization; sexual behavior and history; and substance use (the full survey is included in Supplementary Appendix SA1). The self-sampling survey, which was administered after specimen collection, included questions on the acceptability, comfort, and ease-of-use of the cervicovaginal and anal self-sampling protocols, as well as preference for self- or physician-administered testing/screening.

### Biospecimen collection and processing

Participants were mailed a study test kit prepared by study staff that included materials and instructions for self-collection of three separate biospecimens: a cervicovaginal swab, an anal swab, and an oral rinse. All samples were repackaged in United States Postal Service biospecimen-compliant packaging then returned via mail to the study. Standard laboratory protocols were followed to avoid sample contamination.

#### Cervicovaginal swab

Participants used an Evalyn Brush (Rovers Medical Devices BV, Oss, Netherlands) to self-collect a cervicovaginal sample.^29^ Participants were provided with self-collection instructions that were modified from the original, with permission from Rovers Medical Devices, to remove gendered language. In short, participants were instructed to use the plunger on the device to insert the brush into their vagina to the appropriate depth, rotate the brush five times, and then retract and cap the device for transport. HPV DNA on a dry-stored Evalyn Brush has previously been shown to be analytically stable.^30^

Upon return to the study, the protective cap was removed, and the brush tip was vigorously agitated then gently mashed in a 20 mL vial of PreservCyt solution (Hologic, Inc., Marlborough, MA). The tip of the brush was then removed from the barrel of the device and left to soak for a minimum of 5 minutes to rehydrate dried mucus. After resting, each vial was vortexed for 30 seconds to remove cervical cells that were adhered to the brush bristles. Samples were stored at 0°C.

#### Anal swab

Participants were sent a Dacron flocked swab (Puritan Medical Products, Guilford, ME), a 10 mL conical tube with 1 mL of DNA/RNA Shield (Zymo Research, Irvine, CA) or PreservCyt. Sample collection instructions were based on those developed by Meites et al.^31^; in brief, participants were instructed to insert the swab 2 inches (5 cm) into anus, twirl the swab between the thumb and forefinger while pressing laterally against the anal walls, and then remove the swab from the body. The swab was then placed in the corresponding conical tube. Upon return to the study, the conical tube containing the Dacron flocked swab and transport media were vortexed for 15 seconds to release cells from the fibers of the swab tip. The swab was then extracted from the tube and discarded.

#### Oral rinse

Participants received a 50 mL conical tube with 10 mL Scope Mouthwash (Proctor & Gamble, Cincinnati, OH). Participants were instructed to swish the mouth wash vigorously for 30 seconds, then spit the contents back into the original 50 mL tube.

### HPV testing

#### High-risk HPV testing

Cervicovaginal samples were tested by Michigan Medicine Pathology clinical laboratories using a cobas HR HPV test (Roche Molecular Systems, Inc., Branchburg, NJ), a polymerase chain reaction (PCR)-based test that provides pooled results for genotypes 16, 18, 31, 33, 35, 39, 45, 51, 52, 56, 58, 59, 66, and 68 and individual results for HPV 16 and HPV 18, the highest risk genotypes. The results of this test were returned to participants.

#### PCR mass array HPV genotyping

Cervicovaginal, anal, and oral samples were tested for each of 12 HR (16, 18, 31, 33, 35, 39, 45, 51, 52, 56, 58, 59) and other six HPV genotypes (6, 11, 66, 68, 73, 90); HR was defined by the Carcinogenic [Group 1] designation by the International Agency for Research on Cancer.^32^ Sample processing and DNA extraction details are the same as in Eisenberg et al.,^33^ and technical details of the PCR Mass Array test are given in Walline et al.^34^ Participants whose samples contained insufficient DNA or otherwise resulted in inconclusive test results were denoted as invalid. The results of the PCR Mass Array were not returned to participants.

### Ethics statement

This study was approved by the University of Michigan Institutional Review Board (IRBMED: HUM00166980) and Rogel Cancer Center Protocol Review Committee. Ten TGD community consultants reviewed and commented on the study protocol, surveys, and sample collection instructions; each person was compensated with a $100 gift card. Participants were compensated with a $50 gift card for full study completion and were returned the results of their cervicovaginal cobas HR HPV test, with appropriate cautions given the nonclinical sampling and standard screening recommendations. Because these results depended on the use of a cervicovaginal swab outside of the Food and Drug Administration (FDA) approved context (i.e., used by the participant, not by a physician, to collect a sampling for HPV testing), we applied for and received an FDA determination that the study was a nonsignificant risk device study. Because the anal and oral HPV results were not returned to participants, FDA determination was not needed.

### Statistical analysis

Participant responses were combined in reasonable categories to increase statistical power. Prevalence ratios (PR) for cervicovaginal, oral, and anal HPV as a function of participant characteristics were estimated in log-binomial models in R (R Foundation for Statistical Computing, Vienna, Austria). We calculated concordance of HPV genotypes between cervicovaginal infections and each of oral and anal infections.

## Results

### Demographics, behaviors, and medical history

We enrolled 137 participants in 2020–2021; 125 participants completed the demographic and behavior questionnaire; and 102 participants completed the questionnaire and biospecimen collection. The demographic, behavioral, and medical history characteristics of these 102 participants are summarized in Table 1 (A comparison to the 125 completing the questionnaire are given in Supplementary Table S1.). While participants ranged from 21 to 59 years of age, the majority of those who completed the study (66%) were younger than 30 years old.

**Table 1.** Demographic, Behavioral, and Medical History Characteristics of Participants

	*n*	%
Age group (*N* = 102)		
21–29	67	65.7
30–39	29	28.4
40–59	6	5.9
Race/ethnicity (*N* = 102)		
White	84	82.4
Multiracial	8	7.8
Asian	5	4.9
Black	5	4.9
Gender (*N* = 102)		
Nonbinary, genderfluid, agender	49	48.0
Male, transgender male, transmasculine	50	49.0
Other/prefer not to label	3	2.9
Sexual orientation (*N* = 102)		
Bisexual, pansexual, or omnisexual	44	43.1
Queer	37	36.3
Homosexual	9	8.8
Asexual or demisexual	8	7.8
Straight or heterosexual	3	2.9
Other/prefer not to label	1	1.0
Highest level of education (*N* = 102)		
High school or less	8	7.8
Some college	27	26.5
2- or 4-year degree	46	45.1
Professional or doctorate degree	21	20.6
Current employment status (*N* = 102)		
Full time	38	37.3
Student	22	21.6
Part time	15	14.7
Unemployed, looking for work	12	11.8
Disabled and not working	10	9.8
Unemployed, not looking for work	5	4.9
Yearly household income (*N* = 102)		
Less than $10,000	17	16.8
$10,000–$19,999	19	18.8
$20,000–$39,999	20	19.8
$40,000–$49,999	21	20.8
$50,000+	24	23.8
Developed environment (*N* = 102)		
Suburban	49	48.0
Urban	47	46.1
Rural	6	5.9
Marital status (*N* = 102)		
Single partner, married	47	46.1
Never married or partnered	25	24.5
Single partner, never married	12	11.8
Multiple committed partners	11	10.8
Separated, divorced, or widowed	7	6.9
History of sexual behaviors		
Ever engaged in deep kissing (*N* = 102)	98	96.1
Ever engaged in vaginal/front hole sex (*N* = 97)	95	97.9
Ever engaged in oral sex (*N* = 96)	95	99.0
Ever engaged in insertive anal sex (in either role) (*N* = 95)	57	60.0
Ever engaged in rimming/anilingus (*N* = 100)	43	43.0
Ever engaged in mutual masturbation (*N* = 100)	89	89.0
Gender-affirming transition		
Have transitioned or are transitioning (*N* = 102)	77	75.5
Ever taken hormones for transition (*N* = 76)	58	76.3
Ever had surgery for transitions (*N* = 77)	32	41.6
Substance use (*N* = 100)		
Current alcohol use	75	75.0
Ever cannabis use	54	54.0
Current cannabis use	43	43.0
Ever cigarette use	32	32.0
Current cigarette use	8	8.0
Ever used illegal drugs	21	21.0
Ever illicitly used prescription painkillers	25	25.0
Medical history		
Ever pregnant (*N* = 102)	14	13.7
Ever had a cervical Pap test (*N* = 102)	83	81.4
Ever had an anal Pap test (*N* = 102)	6	5.9
Ever had HPV (*N* = 102)	8	7.8
Ever had other STI (*N* = 100)	22	22.0
At least one dose of HPV vaccine (*N* = 102)	58	56.9
Medical insurance (*N* = 99)		
Private insurance	44	44.4
Public insurance	32	32.3
Parents' private insurance	23	23.2
Ever delayed checkups (*N* = 102)		
Yes, to avoid dysphoria or physical discomfort	61	59.8
Yes, fear of discrimination	16	15.7
Yes, provider lacked knowledge of trans-specific care	6	5.9
No	19	18.6
Ever delayed treatment for injury or illness (*N* = 102)		
Yes, to avoid dysphoria or physical discomfort	37	36.3
Yes, fear of discrimination	14	13.7
Yes, provider lacked knowledge of trans-specific care	3	2.9
No	48	47.1

*N* is the number of participants answering the question, and *n* is the number of respondents answering in the affirmative.

HPV, human papillomavirus; STI, sexually transmitted infection.

There was little racial or ethnic diversity among participants, who were predominantly White (82%). Participants were highly educated, with 92% reporting attending at least some college and 65% having a 2-year, 4-year, or professional/doctorate degree. Reported annual income was distributed fairly evenly between the categories ranging from less than $10,000 to at least $50,000. Most participants reported having a single partner, either married or in committed partnership (46%) or not (12%); about 25% of participants were never married or partnered.

Approximately half (48%) of participants identified as nonbinary, genderfluid, or agender and half (49%) as transgender male, transmasculine, or male (note that people who identify as male and were AFAB may or may not include being transgender as part of their gender identity). Most participants identified their sexual orientation as bisexual, pansexual, or omnisexual (43%) or simply as queer (36%).

The vast majorities of participants reported a history of deep kissing (96%), mutual masturbation (89%), vaginal/front hole sex (98%), and oral sex (99%), while 60% reported ever engaging in anal sex, and 43% having ever engaged in anilingus/rimming (these statistics include both the receptive and performing/penetrating roles.). When asked about partners within the last 6 months, participants overall reported similar numbers of partners with a vagina and partners with a penis for vaginal, anal, or oral sex. A table of recent (last 6 month) sexual activity by sex type and partner genitals is given in Table 2.

**Table 2.** Sexual Behavior History of Participants

	*n*	%
Partners with a vagina, deep kissing, last 6 months (*N* = 99)		
Never	3	3.0
0	44	44.4
1	43	43.4
2–4	9	9.1
5+	0	0.0
Partners with a penis, deep kissing, last 6 months (*N* = 99)		
Never	5	5.1
0	49	49.5
1	33	33.3
2–4	6	6.1
5+	6	6.1
Partners with a vagina, vaginal/oral/anal sex, last 6 months (*N* = 102)		
Never	5	4.9
0	53	52.0
1	38	37.3
2–4	6	5.9
5+	0	0.0
Partners with a penis, vaginal/oral/anal sex, last 6 months (*N* = 102)		
Never	5	4.9
0	54	52.9
1	33	32.4
2–4	7	6.9
5+	3	2.9
Partners with a vagina, vaginal/front hole sex, last 6 months (*N* = 100)		
Never	5	5.0
0	49	49.0
1	41	41.0
2–4	5	5.0
5+	0	0.0
Partners with a penis, vaginal/front hole sex, last 6 months (*N* = 100)		
Never	5	5.0
0	55	55.0
1	28	28.0
2–4	9	9.0
5+	3	3.0
Partners with a vagina, participant receiving oral sex, last 6 months (*N* = 55)		
Never	5	9.1
0	12	21.8
1	31	56.6
2–4	7	12.7
5+	0	0.0
Partners with a penis, participant receiving oral sex, last 6 months (*N* = 44)		
Never	5	11.4
0	3	6.8
1	28	63.6
2–4	5	11.4
5+	3	6.8
Partners with a vagina, participant performing oral sex, last 6 months (*N* = 55)		
Never	5	9.1
0	10	18.2
1	34	61.8
2–4	6	10.9
5+	0	0.0
Partners with a penis, participant performing oral sex, last 6 months (*N* = 44)		
Never	5	11.4
0	2	4.6
1	28	63.6
2–4	6	13.6
5+	3	6.8
Partners with a vagina, anal sex, last 6 months (*N* = 62)		
Never	5	8.1
0	43	69.4
1	14	22.6
2–4	0	0.0
5+	0	0.0
Partners with a penis, anal sex, last 6 months (*N* = 62)		
Never	5	8.1
0	40	64.5
1	14	22.6
2–4	3	4.8
5+	0	0.0

*N* is the number of participants answering the question, and *n* is the number of respondents answering in the affirmative.

About 75% of participants responded that they have transitioned or are currently transitioning from living as the gender assigned at birth, 76% of whom reported ever taking hormones as a part of gender-affirming medical care. Only 42% of transitioned or transitioning participants reported gender-affirming surgery. The vast majority (86%) of participants reported never being pregnant. Most participants indicated currently using alcohol (75%) and marijuana (54%), while only 32% reported ever smoking cigarettes. More than half (57%) reported receiving at least one dose of a vaccination against HPV, and most (81%) reported having ever had a cervical pap smear.

While none of the participants reported being uninsured, 81% reported delaying regular medical checkups. Most of these (59%) were due to fear of physical discomfort or dysphoria. Most participants (53%) also reported delaying care for acute medical needs, with 36% of participants reporting delaying acute treatment due to concerns for physical discomfort or dysphoria.

### HPV prevalence and determinants

**FIG. 1.**
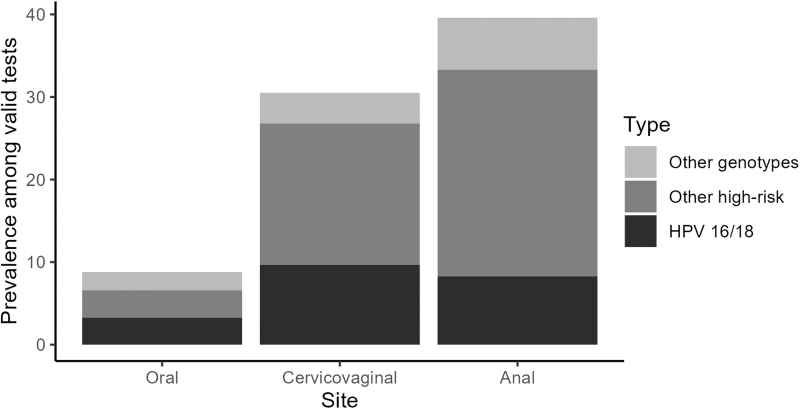
Prevalence of oral, cervicovaginal, and anal HPV by genotype category. The genotypes in the “Other high-risk” HPV category were 31, 33, 35, 39, 45, 51, 52, 56, 58, and 59, and the “Other genotypes” were 6, 11, 66, 68, 73, and 90. HPV, human papillomavirus.

HPV was detected in 8.8% of 91 valid tests on oral samples, 30.5% of 82 valid tests on cervicovaginal samples, and 39.6% of 48 valid tests on anal samples (Fig. 1). HR HPV types were detected in 6.6% of oral, 26.8% of cervicovaginal, and 33.3% of anal samples. Genotypes 16 and 18 were detected in 3.3% of oral, 9.7% of cervicovaginal, and 8.3% of anal samples. Genotype-specific HPV prevalence by site is given in Supplementary Table S2. Of the 41 participants who had a valid sample at all three sites, 56.1% were positive for at least one HPV genotype in at least one anatomical site, 53.6% were positive for at least one HR genotype, and 22.0% were positive for genotypes 16 or 18.

Of the 44 participants with both valid cervicovaginal and anal test results, 20.5% (9) of pairs of anal and cervicovaginal test results detected the presence of HPV of the same genotype. These concordant infections represented 56.3% of 16 cervicovaginal infections in this group and 50.0% of 18 anal infections. Of the 75 participants with valid results for both oral and cervicovaginal tests, 6.7% (5) of pairs of oral and cervicovaginal tests detected the presence of the same genotype of HPV. These concordant infections represented only 20.8% of 24 cervicovaginal infections in this group but 71.4% of 7 oral infections. The HPV genotype-specific concordance for anal/cervicovaginal and oral/cervicovaginal samples are given in Supplementary Table S3.

HPV prevalence varied across oral, cervicovaginal, and anal sites and across demographic and behavioral characteristics (Supplementary Table S4). No statistically significant associations were found between any characteristic and oral HPV infection. Cervicovaginal HPV infection was statistically significantly associated with the number of deep kissing partners with a penis in the last 6 months (PR = 3.12 [95% CI = 1.56–6.23] for 5+ partners vs. 0 partners), number of partners with a vagina from whom the participant received oral sex (PR = 3.47 [95% CI = 1.52–7.93] for 2–4 partners vs. 0 partners), a history of HPV (PR = 2.68 [95% CI = 1.47–4.88]), and a history of other STI (PR = 2.07 [95% CI = 1.11–3.85]).

Anal HPV infection was statistically significantly associated with sexual orientation (PR = 1.94 [95% CI = 1.06–3.58] for identifying as homosexual vs. bisexual, pansexual, or omnisexual), number of deep kissing partners with a penis in the last 6 months (PR = 6.00 [95% CI = 1.54–23.4] for 5+ partners vs. 0 partners), and a history of other STI (PR = 2.67 [95% CI = 1.33–5.36]).

## Discussion

We evaluated the prevalence of cervicovaginal, oral, and anal HPV in a Midwestern TGD AFAB population and investigated the potential association of infection with demographics, health factors, and sexual behaviors. Our participants were overall young (66% under 30) and sexually active (with the vast majority having ever received or performed vaginal and oral sex), with about half of the sample identifying as male or transgender male and half as nonbinary or another gender diverse identity.

Although some physicians and patients believe that TGD people AFAB are at low risk for HPV infection,^4^ HR HPV was not uncommon among our TGD AFAB participants. We found HPV prevalence of 8.8% (HR = 6.6%; HPV 16/18 = 3.3%) in oral samples, 30.5% (HR = 26.8%; HPV 16/18 = 9.7%) in cervicovaginal samples, and 39.6% (HR = 33.3%; HPV 16/18 = 8.3%) in anal samples. Because HR HPV can lead to cancer, it is important that screening for cervicovaginal HPV (and perhaps other sites in the future) is accessible by TGD people AFAB through affirming medical providers, or, potentially, at-home self-screening.

A separate study in a TGD AFAB population in Boston, Massachusetts, found 16.0% HR HPV prevalence in physician-collected cervicovaginal swabs and 13.0% in self-collected swabs,^13^ compared to the 26.8% HR HPV prevalence in our study. In our partner study using largely similar protocols for oral and cervicovaginal HPV detection in a general population with a similar age profile, 5.3% of participants (cisgender men and women combined) for HR oral HPV, and 4.6% of cisgender women were positive for HR cervicovaginal HPV (anal swabs were not collected in the partner study).^35^

Nationally representative data (2009–2014) have estimated HR cervicovaginal HPV prevalence at 18.6%, HR oral HPV prevalence in cisgender women as 1.3%, and HR oral HPV prevalence in cisgender men at 5.6%, although caution should be taken in comparing our study to these data because of the different age profiles and other demographics.^36^ Overall, these studies suggest that there is little evidence that TGD people AFAB are at lower risk for HPV and HPV-related cancers than cisgender people.

We found a high prevalence of anal HPV among valid tests (39.6%). A meta-analysis of anal HPV prevalence in cisgender women found that cisgender women without HPV-associated cervical pathology had anal HPV prevalence ranging from 4% to 22%, and prevalence in those with HPV-related cervical pathology ranged from 23% to 36%.^37^ A separate analysis of participants' experiences with the cervicovaginal and anal sample self-collection found that some participants reported difficulty using the swabs because of the size or shape of their bodies.^38^

We found significant differences in HPV prevalence across several participant characteristics and reported behavior. History of an STI diagnosis, likely a proxy for unmeasured behavior, was also associated with HPV at both cervicovaginal (PR = 2.07, 95% CI = 1.11–3.85) and anal (PR = 2.67, 95% CI = 1.33–5.36) sites. HPV is thought to be largely transmitted through sexual activity, and many previous studies have demonstrated associations in general populations.^39–45^ Few associations rose to the level of statistical significance, with the exception of the number of deep kissing partners with a penis for both cervicovaginal and anal HPV infection and the number of partners with a vagina that a participant received oral sex from for cervicovaginal HPV infection. More broadly, in many sexual behavior categories, prevalence generally increased with the number of recent sexual partners, even if not statistically significantly.

In addition, although the effects were not statistically significant, there were indications that HPV prevalence among the participants identifying as male, transgender male, or transmasculine was higher than among those identifying as nonbinary, genderfluid, or agender. There were no significant differences in HPV prevalence by transition status, use of gender-affirming hormones, or gender-affirming surgery.

We found a high degree of cervicovaginal–anal concordance among the participants with valid results for both anal and cervicovaginal tests, highlighting the need to consider multiple anatomical sites simultaneously. We cannot tell from our data whether this correlation is the result of autoinoculation from site to site or separate transmission events from the same partner to different sites. We found that many oral infections were genotype concordant with cervicovaginal infections (71%), but few cervicovaginal infections were concordant with an oral infection (21%). These results are consistent with patterns of oral-cervicovaginal concordance in the National Health and Nutrition Examination Survey.^36^ Together, our results are consistent with cervicovaginal infections being the source of anal and oral infections.

### Strengths and limitations

One strength of our study is the multiple anatomical site design, incorporating oral, cervicovaginal, and anal self-sampling and HPV testing. In addition, because many gender minority health studies in the United States are focused on urban, coastal populations, this Midwestern cohort helps to bring context and enhance the generalizability of the literature. We also explicitly included nonbinary and gender diverse participants in this study, who are often overlooked in gender minority research.

One limitation of our study is the small sample size, limiting statistical power, although other, comparable studies have also had limited sample sizes.^13^ Moreover, lack of racial and ethnic diversity in this pilot study is a strong limitation, as there may be important intersectional effects for TGD people of color. Indeed, we found high HPV prevalence among the participants of color in this study. Future work should use targeted recruitment strategies to achieve better racial and ethnic representation.

Another limitation was the low sample validity (i.e., low detection of human control indicators) in our anal samples (47% valid). Validity was much higher among oral (89% valid) and cervicovaginal (80% valid) samples. We are uncertain whether some participants may have not collected the samples or there may have been an unidentified problem with the provided supplies or sampling instructions. Future work could involve instructional videos demonstrating sample collection techniques to increase participants' confidence in and adherence to the protocol.

Finally, while the age distribution of the cohort (66% aged <30 years) is not inherently a limitation, it should be accounted for when generalizing the results, since HPV prevalence varies by age,^35^ possibly as a function of the number of recent and lifetime sexual partnerships.

## Conclusion

We detected HR cervicovaginal, oral, and anal HPV in TGD people AFAB. We found HPV oral prevalence consistent with cisgender populations but found higher prevalence of cervicovaginal and anal infection, suggesting that there is little evidence that TGD people AFAB are at lower risk for HPV and HPV-related cancers than cisgender people. It is essential that we reduce barriers to cancer screening for TGD populations. Future work is needed to support the development and validation of HPV testing of self-collected specimens (for both cisgender and TGD people AFAB), as well as the creation of TGD-specific and inclusive guidelines, instructions, and options for cervical cancer screening.

## Notes

Supplementary Appendix SA1

## References

[1] Centers for Disease Control and Prevention. Cancers associated with human papillomavirus, United States, 2011 to 2015. United States Cancer Statistics Data Brief, No. 4. Centers for Disease Control and Prevention: Atlanta, GA; 2018.

[2] Curry SJ, Krist AH, Owens DK, et al. Screening for cervical cancer. JAMA 2018;320(7):674–686; doi: 10.1001/jama.2018.10897 30140884

[3] Reisner SL, Murchison GR. A global research synthesis of HIV and STI biobehavioural risks in female-to-male transgender adults. Glob Public Health 2016;11(7–8):866–887; doi: 10.1080/17441692.2015.1134613 26785800 PMC4993101

[4] Agenor M, Peitzmeier SM, Bernstein IM, et al. Perceptions of cervical cancer risk and screening among transmasculine individuals: Patient and provider perspectives. Cult Health Sex 2016;18(10):1192–1206; doi: 10.1080/13691058.2016.1177203 27142466

[5] Peitzmeier SM, Reisner SL, Harigopal P, et al. Female-to-male patients have high prevalence of unsatisfactory Paps compared to non-transgender females: Implications for cervical cancer screening. J Gen Intern Med 2014;29(5):778–784; doi: 10.1007/s11606-013-2753-1 24424775 PMC4000345

[6] Gatos KC. A literature review of cervical cancer screening in transgender men. Nurs Womens Health 2018;22(1):52–62; doi: 10.1016/j.nwh.2017.12.008 29433700

[7] Obedin-Maliver J, de Haan G. Gynecologic care for transgender adults. Curr Obstet Gynecol Rep 2017;6(2):140–148; doi: 10.1007/s13669-017-0204-4

[8] Seay J, Ranck A, Weiss R, et al. Understanding transgender men's experiences with and preferences for cervical cancer screening: A rapid assessment survey. LGBT Health 2017;4(4):304–309; doi: 10.1089/lgbt.2016.0143 28422558

[9] Potter J, Peitzmeier SM, Bernstein I, et al. Cervical cancer screening for patients on the female-to-male spectrum: A narrative review and guide for clinicians. J Gen Intern Med 2015;30(12):1857–1864; doi: 10.1007/s11606-015-3462-8 26160483 PMC4636588

[10] Peitzmeier SM, Khullar K, Reisner SL, et al. Pap test use is lower among female-to-male patients than non-transgender women. Am J Prev Med 2014;47(6):808–812; doi: 10.1016/j.amepre.2014.07.031 25455121

[11] Dhillon N, Oliffe JL, Kelly MT, et al. Bridging barriers to cervical cancer screening in transgender men: A scoping review. Am J Mens Health 2020;14(3):1557988320925691; doi: 10.1177/1557988320925691 32489142 PMC7271678

[12] Kattari SK, Gross EB, Harner V, et al. “Doing it on my own terms”: Transgender and nonbinary adults' experiences with HPV self-swabbing home testing kits. Womens Reprod Health (Phil) 2023;10(4):496–512; doi: 10.1080/23293691.2022.2094737 PMC10720596 38105788

[13] Reisner SL, Deutsch MB, Peitzmeier SM, et al. Test performance and acceptability of self-versus provider-collected swabs for high-risk HPV DNA testing in female-to-male trans masculine patients. PLoS One 2018;13(3):e0190172; doi: 10.1371/journal.pone.0190172 29538411 PMC5851532

[14] Reisner SL, Deutsch MB, Peitzmeier SM, et al. Comparing self- and provider-collected swabbing for HPV DNA testing in female-to-male transgender adult patients: A mixed-methods biobehavioral study protocol. BMC Infect Dis 2017;17:444; doi: 10.1186/s12879-017-2539-x 28645254 PMC5481878

[15] McDowell M, Pardee DJ, Peitzmeier S, et al. Cervical cancer screening preferences among trans-masculine individuals: Patient-collected human papillomavirus vaginal swabs versus provider-administered Pap tests. LGBT Health 2017;4(4):252–259; doi: 10.1089/lgbt.2016.0187 28665783

[16] Maza M, Meléndez M, Herrera A, et al. Cervical cancer screening with human papillomavirus self-sampling among transgender men in El Salvador. LGBT Health 2020;7(4):174–181; doi: 10.1089/lgbt.2019.0202 32407149 PMC7301324

[17] Brown B, Poteat T, Marg L, et al. Human papillomavirus-related cancer surveillance, prevention, and screening among transgender men and women: Neglected populations at high risk. LGBT Health 2017;4(5):315–319; doi: 10.1089/lgbt.2016.0142 28876211

[18] Jemal A, Simard EP, Dorell C, et al. Annual report to the nation on the status of cancer, 1975–2009, featuring the burden and trends in human papillomavirus (HPV)-associated cancers and HPV vaccination coverage levels. J Natl Cancer Inst 2013;105(3):175–201; doi: 10.1093/jnci/djs491 23297039 PMC3565628

[19] Siegel RL, Miller KD, Jemal A. Cancer statistics, 2018. CA Cancer J Clin 2018;68(1):7–30; doi: 10.3322/caac.21442 29313949

[20] Van Dyne EA, Henley SJ, Saraiya M, et al. Trends in human papillomavirus–associated cancers—United States, 1999–2015. MMWR Morb Mortal Wkly Rep 2018;67(33):918–924; doi: 10.15585/mmwr.mm6733a2 30138307 PMC6107321

[21] Steinau M, Hariri S, Gillison ML, et al. Prevalence of cervical and oral human papillomavirus infections among US women. J Infect Dis 2014;209(11):1739–1743; doi: 10.1093/infdis/jit799 24319284 PMC4122915

[22] Brouwer AF, Eisenberg MC, Carey TE, et al. Trends in HPV cervical and seroprevalence and associations between oral and genital infection and serum antibodies in NHANES 2003–2012. BMC Infect Dis 2015;15:575; doi: 10.1186/s12879-015-1314-0 26689203 PMC4687319

[23] Kedarisetty S, Orosco RK, Hecht AS, et al. Concordant oral and vaginal human papillomavirus infection in the United States. JAMA Otolaryngol Head Neck Surg 2016;142(5):457–465; doi: 10.1001/jamaoto.2016.0064 27010384

[24] Brouwer AF, Meza R, Eisenberg MC. Transmission heterogeneity and autoinoculation in a multisite infection model of HPV. Math Biosci 2015;270(Pt A):115–125; doi: 10.1016/j.mbs.2015.10.012 26518265 PMC4677058

[25] Patel EU, Rositch AF, Gravitt PE, et al. Concordance of penile and oral human papillomavirus infections among men in the United States. J Infect Dis 2017;215(8):1207–1211; doi: 10.1093/infdis/jix116 28329127 PMC5441108

[26] Sonawane K, Suk R, Chiao EY, et al. Oral human papillomavirus infection: Differences in prevalence between sexes and concordance with genital human papillomavirus infection, NHANES 2011 to 2014. Ann Intern Med 2017;167(10):714–724; doi: 10.7326/M17-1363 29049523 PMC6203692

[27] Blas MM, Brown B, Menacho L, et al. HPV prevalence in multiple anatomical sites among men who have sex with men in Peru. PLoS One 2015;10(10):e0139524; doi: 10.1371/journal.pone.0139524 26437318 PMC4593601

[28] King EM, Gilson R, Beddows S, et al. Oral human papillomavirus (HPV) infection in men who have sex with men: Prevalence and lack of anogenital concordance. Sex Transm Infect 2015;91(4):284–286; doi: 10.1136/sextrans-2014-051955 25887283 PMC4453633

[29] Rovers Medical Devices. Evalyn^®^ Brush. Oss, the Netherlands; 2022. Available from: www.roversmedicaldevices.com/cell-sampling-devices/evalyn-brush/ [Last accessed: September 12, 2023].

[30] Ejegod DM, Pedersen H, Alzua GP, et al. Time and temperature dependent analytical stability of dry-collected Evalyn HPV self-sampling brush for cervical cancer screening. Papillomavirus Res 2018;5:192–200; doi: 10.1016/j.pvr.2018.04.005 29689311 PMC6026099

[31] Meites E, Gorbach PM, Gratzer B, et al. Monitoring for human papillomavirus vaccine impact among gay, bisexual, and other men who have sex with men—United States, 2012–2014. J Infect Dis 2016;214(5):689–696; doi: 10.1093/infdis/jiw232 27296847 PMC5049501

[32] Bouvard V, Baan R, Straif K, et al. A review of human carcinogens—Part B: Biological agents. Lancet Oncol 2009;10(4):321–322; doi: 10.1016/s1470-2045(09)70096-8 19350698

[33] Eisenberg MC, Campredon LP, Brouwer AF, et al. Dynamics and determinants of HPV infection: The Michigan HPV and Oropharyngeal Cancer (M-HOC) Study. BMJ Open 2018;8(10):e021618; doi: 10.1136/bmjopen-2018-021618 PMC6169774 30282679

[34] Walline HM, Komarck C, McHugh JB, et al. High-risk human papillomavirus detection in oropharyngeal, nasopharyngeal, and oral cavity cancers: Comparison of multiple methods. JAMA Otolaryngol Head Neck Surg 2013;139(12):1320–1327; doi: 10.1001/jamaoto.2013.5460 24177760 PMC4049419

[35] Brouwer AF, Campredon LP, Walline HM, et al. Prevalence and determinants of oral and cervicogenital HPV infection: Baseline analysis of the Michigan HPV and Oropharyngeal Cancer (MHOC) cohort study. PLoS One 2022;17(5):e0268104; doi: 10.1371/journal.pone.0268104 35576195 PMC9109914

[36] Brouwer AF, Eisenberg MC, Carey TE, et al. Multisite HPV infections in the United States (NHANES 2003–2014): An overview and synthesis. Prev Med 2019;123:288–298; doi: 10.1016/j.ypmed.2019.03.040 30959071 PMC6534472

[37] Stier EA, Sebring MC, Mendez AE, et al. Prevalence of anal human papillomavirus infection and anal HPV-related disorders in women: A systematic review. Am J Obstet Gynecol 2015;213(3):278–309; doi: 10.1016/j.ajog.2015.03.034 25797230 PMC4556545

[38] Welsh EF, Andrus EC, Sandler CB, et al. Cervicovaginal and anal self-sampling for HPV testing in a transgender and gender diverse population assigned female at birth: Comfort, difficulty, and willingness to use. [Preprint] medRxiv 2023.08.15.23294132; doi: 10.1101/2023.08.15.23294132 38574315

[39] Cook RL, Thompson EL, Kelso NE, et al. Sexual behaviors and other risk factors for oral human papillomavirus infections in young women. Sex Transm Dis 2014;41(8):486–492; doi: 10.1097/OLQ.0000000000000159 25013976 PMC5528151

[40] Pickard RK, Xiao W, Broutian TR, et al. The prevalence and incidence of oral human papillomavirus infection among young men and women, aged 18–30 years. Sex Transm Dis 2012:39(7):559–566; doi: 10.1097/OLQ.0b013e31824f1c65 22706220

[41] Brouwer AF, Campredon LP, Walline HM, et al. Incidence and clearance of oral and cervicogenital HPV infection: Longitudinal analysis of the MHOC cohort study. BMJ Open 2022;12(1):e056502; doi: 10.1136/bmjopen-2021-056502 PMC8724815 34980629

[42] Edelstein ZR, Schwartz SM, Hawes S, et al. Rates and determinants of oral human papillomavirus (HPV) infection in young men. Sex Transm Dis 2012;39(11):860–867; doi: 10.1097/OLQ.0b013e318269d098 23064535 PMC4375438

[43] D'Souza G, Cullen K, Bowie J, et al. Differences in oral sexual behaviors by gender, age, and race explain observed differences in prevalence of oral human papillomavirus infection. PLoS One 2014;9(1):e86023; doi: 10.1371/journal.pone.0086023 24475067 PMC3901667

[44] D'Souza G, Wentz A, Kluz N, et al. Sex differences in risk factors and natural history of oral human papillomavirus infection. J Infect Dis 2016;213(12):1893–1896; doi: 10.1093/infdis/jiw063 26908748 PMC9136851

[45] Chung CH, Bagheri A, D'Souza G. Epidemiology of oral human papillomavirus infection. Oral Oncol 2014;50(5):364–369; doi: 10.1016/j.oraloncology.2013.09.003 24080455 PMC3968244

