# Supplementary material for: Prevalence and Determinants of Cervicovaginal, Oral, and Anal Human Papillomavirus Infection in a Population of Transgender and Gender Diverse People Assigned Female at Birth
Source: LGBT Health. 2024 Sep 5;11(6):437–45. doi: 10.1089/lgbt.2023.0335 (PMC11449398; doi:10.1089/lgbt.2023.0335)
Supplement: Supplementary Appendix SA1 [file lgbt.2023.0335_suppl_appendixsa1.pdf]

Supplementary Appendix SA1

Transmasculine and Nonbinary Demographic and Behavior Survey

Start of Block: Consent and ID Number

JS

Q1 Thank you for taking the time to participate in our research project. This survey will take at most 60 minutes to complete, and you may skip any question for any reason. We will be asking you questions about your personal characteristics, sexual healthcare, sexual behavior, sexual partners, and substance use/abuse among other things, and we understand these are private topics. All answers you provide are confidential and will be used only for research purposes.

We solicited feedback from trans and nonbinary community members on this survey, and we have made our best attempts to use representative and respectful language. We apologize in advance if any questions miss the mark.

Please press the next button when you are ready to begin the survey. Please note that you may not always be able to go backward in the survey after some questions, so please enter your answers carefully.

Page Break

Q2
ID Number

Please enter your participant ID.

____

Page Break

End of Block: Consent and ID Number

Start of Block: Demographic Questions

Q3
Demographic Questions

We are now going to ask you some basic questions about your personal and social background.

What is your gender?

____

Q4 **Gender is complicated. For the purposes of statistical analysis, which of the following most closely matches the answer you gave above?**

○ Male (1)

○ Female (2)

○ Transgender male/Transmasculine (3)

○ Transgender female/Transfeminine (4)

○ Nonbinary, genderqueer, or a related or more specific term outside of “man” or “woman” (e.g agender, genderfluid, bigender) (5)

○ Another gender not listed (6)

○ Prefer not to label (7)

Q5
What sex were you assigned at birth (i.e., on your original birth certificate)?

- ○ Male (1)
- ○ Female (2)

Q6 What is your sexual orientation?

____

X→

Q7 **Sexual orientation is also complicated.** For the purposes of statistical analysis, which of the following most closely matches the answer you gave above?
We understand that some of these categories may overlap, so pick the one that is the closest match.

- ○ Straight/heterosexual (1)
- ○ Gay/lesbian (2)
- ○ Bisexual, pansexual, or omnisexual (3)
- ○ Asexual/demisexual (4)
- ○ Queer (7)
- ○ Another orientation not listed (5)
- ○ Prefer not to label (8)

Page Break

Q8 **What is your birth year?**

(Please enter in YYYY format.)

____

Q9 **Which of the following ethnic or cultural groups are you a member of?**

You may choose more than one group.

- ☐ Indigenous American (A person having origins in any of the original peoples of North and South America (including Central America), and who maintains a tribal affiliation or community attachment.) (1)
- ☐ Asian (A person having origins in any of the original peoples of the Far East, Southeast Asia, or the Indian subcontinent including, for example, Cambodia, China, India, Japan, Korea, Malaysia, Pakistan, the Philippine Islands, Thailand, and Vietnam.) (2)
- ☐ Black, African American, or African (A person having origins in any of the Black racial groups of Africa – includes Caribbean Islanders and other of African origin.) (3)
- ☐ Native Hawaiian or Other Pacific Islander (A person having origins in any of the original peoples of Hawaii, Guam, Samoa, or other Pacific Islands.) (4)
- ☐ White (A person having origins in any of the original peoples of Europe, the Middle East, or North Africa.) (5)
- ☐ Hispanic or Latina/o/x (A person of Cuban, Mexican, Puerto Rican, South or Central American, or other Spanish culture or origin, regardless of race.) (6)
- ☐ Other (7) ____

Page Break

Q10 **What is your weight in pounds (lbs)?**

We are asking because people with different body types may have different experiences with the test procedures.

____

*

Q11 **What is your height in feet and inches?**

	Feet (1)	Inches (2)
Height (4)		

Page Break

Q12 **What is the highest level of education that you have completed?**

- Less than high school (1)
- High school graduate (2)
- Some college (3)
- 2 year degree (4)
- 4 year degree (5)
- Professional degree (6)
- Doctorate (7)

Q13 **What is your employment status?**

- Employed full time (11)
- Employed part time (12)
- Unemployed looking for work (13)
- Unemployed not looking for work (14)
- Retired (15)
- Student (16)
- Disabled and not working (17)

Q14 **What is your yearly household income?**

- Less than $10,000 (13)
- $10,000 - $19,999 (14)
- $20,000 - $29,999 (15)
- $30,000 - $39,999 (16)
- $40,000 - $49,999 (17)
- $50,000 - $59,999 (18)
- $60,000 - $69,999 (19)
- $70,000 - $79,999 (20)
- $80,000 - $89,999 (21)
- $90,000 - $99,999 (22)
- $100,000 - $149,999 (23)
- More than $150,000 (24)

Page Break

Q15 **What is your current marital or partner status?**

- ○ Never been married or partnered (1)
- ○ Single partner, not married (3)
- ○ Single partner, married (2)
- ○ Multiple committed partners (4)
- ○ Formerly but not currently partnered (separated/divorced/widowed) (5)

Q16 How would you describe the community that you currently reside in?

- ○ Rural (1)
- ○ Suburban (2)
- ○ Urban (4)

End of Block: Demographic Questions

Start of Block: Healthcare Utilization Questions

Q17 Healthcare Utilization Questions
The following questions are about your use of healthcare.

When was the last time you saw a medical professional, such as in a primary care, specialist, or urgent care setting?

- ◯ Withing the last 6 months (1)
- ◯ 6 months to less than 1 year ago (2)
- ◯ 1 year to less than 2 years ago (3)
- ◯ 2 years to less than 5 years ago (4)
- ◯ More than 5 years ago (5)

Q18 **What kind of medical insurance do you currently have?**

- ◯ Public insurance (e.g., Medicaid, Medicare) (1)
- ◯ Private insurance (including insurance through a college/university) (2)
- ◯ Parent's private insurance (3)
- ◯ No insurance (4)
- ◯ Unsure (5)

Q19 **Have you ever delayed preventive care, such as annual check-ups, because of fear of discrimination, lack of provider knowledge, or expectation of physical discomfort or dysphoria?**
Select all that apply.

- [ ] Yes, for fear of discrimination (1)
- [ ] Yes, because the provider lacked knowledge of trans* care (0)
- [ ] Yes, because I wanted to avoid dysphoria or physical discomfort (2)
- [ ] ⊗No (3)

Q20 **Have you ever delayed care when sick or injured because of fear of discrimination,** lack of provider knowledge, or expectation of physical discomfort or dysphoria?

- [ ] Yes, for fear of discrimination (1)
- [ ] Yes, because the provider lacked knowledge of trans* care (0)
- [ ] Yes, because I wanted to avoid dysphoria or physical discomfort (2)
- [ ] ⊗No (3)

End of Block: Healthcare Utilization Questions

Start of Block: Gender-Affirming Care Questions

Q21 Gender-Affirming Care Questions
The following questions are about your use of gender-affirming care.

Have you transitioned or are you transitioning from living as the gender assigned to you at birth? This transition may or may not include medical interventions.

- ○ Yes (1)
- ○ No (0)

Page Break

Display This Question:

If Gender-Affirming Care Questions The following questions are about your use of gender-affirming c... = Yes

Q22 **At what age did you transition, or begin to transition?**

Age:

____

Display This Question:

If Gender-Affirming Care Questions The following questions are about your use of gender-affirming c... = Yes

Q23 **Have you ever used hormones as part of a gender-affirming transition?**

- Yes (1)
- No (0)

Display This Question:

If Have you ever used hormones as part of a gender-affirming transition? = Yes

Q24 **Have you ever used gender-affirming hormones that were not prescribed by a healthcare professional?**

- Yes (1)
- No (0)

Display This Question:

If Have you ever used hormones as part of a gender-affirming transition? = Yes

Q25 **Are you currently using gender-affirming hormones?**

- Yes (1)
- No (0)

Display This Question:

If Are you currently using gender-affirming hormones? = Yes

Q26 **How long have you been consistently using gender-affirming hormones?**
By "consistently," we mean without stopping use; missed doses are okay.

- Less than 6 months (1)
- 6 months to less than 1 year (2)
- 1 year to less than 3 years (3)
- 3 years to less than 5 years (4)
- More than 5 years (5)

Display This Question:

If Gender-Affirming Care Questions The following questions are about your use of gender-affirming c... = Yes

Q27 **Have you ever had surgery as part of a gender-affirming transition?**

- Yes (1)
- No (0)

Display This Question:

If Have you ever had surgery as part of a gender-affirming transition? = Yes

Q28 **Which gender-affirming surgeries have you had?**

- ☐ Chest surgery (FTM reconstruction/bilateral mastectomy) (1)
- ☐ Chest surgery (breast reduction without breast removal) (2)
- ☐ Facial or neck surgery (3)
- ☐ Oophorectomy (removal ofboth ovaries and fallopian tubes) (4)
- ☐ Partial or supracervical hysterectomy (removal of uterus, cervix intact) (5)
- ☐ Total hysterectomy (removal of uterus and cervix) (6)
- ☐ Vaginectomy (removal of vagina) (9)
- ☐ Metoidioplasty or phallorplasty genital surgery (formation of a new penis) (7)
- ☐ Other (8) ____

Page Break

End of Block: Gender-Affirming Care Questions

Start of Block: Sexual Health Questions

Q29
Sexual Health Questions
The following questions are about your sexual healthcare.

Have you ever been diagnosed with a sexually transmitted infection (STI)? If so, which ones?

- ☐ ⊗ Never been diagnosed with an STI (1)
- ☐ HIV (human immunodeficiency virus) (2)
- ☐ HPV (human papillomavirus) (3)
- ☐ Chlamydia (4)
- ☐ Gonorrhea (5)
- ☐ Syphillis (6)
- ☐ Hepatitis B or C (7)
- ☐ Trichomoniasis (trich) (8)
- ☐ Herpes (9)
- ☐ Other (10) ____

Page Break

Q30

Have you ever had a yeast infection?

○ Yes (1)

○ No (0)

Page Break

Display This Question:

If Have you ever had a yeast infection? = Yes

*

Q31 **Approximately how old were you at your last yeast infection?**

Age:

____

Page Break

Display This Question:

If What sex were you assigned at birth (i.e., on your original birth certificate)? != Male

Q32 **Have you ever had a genital infection other than a yeast infection?**

- Yes (1)
- No (0)

Page Break

Display This Question:

If Have you ever had a genital infection other than a yeast infection? = Yes

*

Q33 **Approximately how old were you at your last genital infection other than a yeast infection?**

Age:

____

Page Break

Q34 **Have you ever had a urinary tract infection (UTI)?**

- ○ Yes (1)
- ○ No (0)

Page Break

Display This Question:

If Have you ever had a urinary tract infection (UTI)? = Yes

*

Q35 **Approximately how old were you at your last UTI?**

Age:

____

Page Break

Display This Question:

If What sex were you assigned at birth (i.e., on your original birth certificate)? != Male

Q36

Have you ever had a cervical pap smear?

- Yes (1)
- No (0)

Page Break

Display This Question:

If Have you ever had a cervical pap smear? = Yes

*

Q37

Approximately how old were you at your last cervical pap smear?

Age:

____

Page Break

Display This Question:

If Have you ever had a cervical pap smear? = Yes

Q38 **Have you ever had an abnormal or inadequate cervical pap smear?**

- Yes, abnormal (1)
- Yes, inadequate (2)
- No (0)

Page Break

Display This Question:

If Have you ever had an abnormal or inadequate cervical pap smear? = Yes, abnormal

Q39 **Approximately how old were you at your last abnormal or inadequate cervical pap smear?**

Age:

____

Display This Question:

If Have you ever had an abnormal or inadequate cervical pap smear? = Yes, abnormal

Q40 **Has a doctor every recommended you have surgical treatment (e.g., LEEP, cone biopsy, cryotherapy) as a result of an abnormal cervical pap smear?**

- ◯ Yes, and I had the surgical treatment (1)
- ◯ Yes, but I did not have the surgical treatment (2)
- ◯ No, surgical treatment was not recommended (0)

Page Break

Display This Question:

If What sex were you assigned at birth (i.e., on your original birth certificate)? != Male

Q41 **Have you ever had a genital HPV test?** An HPV test is a genital swab to test for the presence of the virus.

- Yes (1)
- No (0)
- Unsure (2)

Page Break

Display This Question:

If Have you ever had a genital HPV test? An HPV test is a genital swab to test for the presence of... = Yes

*

Q42 **How old were you at your last genital HPV test?** If unsure, please provide your best estimate.

Age:

____

Page Break

Display This Question:

If Have you ever had a genital HPV test? An HPV test is a genital swab to test for the presence of... = Yes

X→

Q43 **Has your genital HPV test ever come back positive for high-risk HPV?**

- Yes (1)
- No (0)
- Unsure (2)

Page Break

Display This Question:

If Has your genital HPV test ever come back positive for high-risk HPV? = Yes

Q44 **How old were when your genital HPV came back positive for high-risk HPV most recently?** If unsure, please provide your best estimate.

Age:

____

Page Break

Q45 **Have you ever had an anal pap smear?**

- Yes (1)
- No (0)

Page Break

Display This Question:

If Have you ever had an anal pap smear? = Yes

Q46 **How old were you at your last anal pap smear?** If unsure, please provide your best estimate.

Age:

____

End of Block: Sexual Health Questions

Start of Block: Sexual Health & Vaccinations - Overview

Q47

Have you been vaccinated against human papillomavirus (HPV)?

- Yes (1)
- No (0)

Display This Question:

If Have you been vaccinated against human papillomavirus (HPV)? = Yes

Q48 **How many doses of human papillomavirus (HPV) vaccine did you receive?**

- 1 (1)
- 2 (2)
- 3 (3)
- Don't know (4)

Page Break

Display This Question:

If Have you been vaccinated against human papillomavirus (HPV)? = Yes

*

Q49 **What age did you get your first dose of HPV vaccine?**

Age:

____

Page Break

Q50 **Have you ever been pregnant?**

- Yes (1)
- No (0)

Display This Question:

If Have you ever been pregnant? = Yes

Q51 **How many children have you given birth to?**

____

End of Block: Sexual Health & Vaccinations - Overview

Start of Block: Kissing

Q52 The next set of questions is about deep kissing behavior. By deep kissing, we mean open-mouth or "French kissing" involving tongue-to-tongue contact for a prolonged period of time. A deep kissing partner is defined as a person with whom you engage in deep kissing.

Have you ever had any deep kissing partners?

- Yes (1)
- No (0)

Skip To: End of Block If The next set of questions is about deep kissing behavior. By deep kissing, we mean open-mouth or... != Yes

---

Page Break

Q53

A deep kissing partner is defined as a person with whom you engage in deep kissing. By deep kissing, we mean open-mouth or "French kissing" involving tongue-to-tongue contact for a prolonged period of time.

Because transmission of the human papillomavirus may depend on one's genitals, throughout these sections we are going ask you to categorize your partners by their genitals, regardless of their gender. We understand that this is reductive, but we believe that this is the most scientifically relevant distinction. If you do not know your deep kissing partner's genitals, please classify the partner as you think is most appropriate.

Approximately how many deep kissing partners with a vagina/front hole have you had in the LAST SIX MONTHS?

Number:

____

Q54 **Approximately how many deep kissing partners with a vagina/front hole have you had in the LAST YEAR?**

Number:

____

Q55 **Approximately how deep kissing partners with a vagina/front hole have you had in your LIFETIME?**
Number:

____

Page Break

Q56

A deep kissing partner is defined as a person with whom you engage in deep kissing. By deep kissing, we mean open-mouth or "French kissing" involving tongue-to-tongue contact for a prolonged period of time.

Because transmission of the human papillomavirus may depend on one's genitals, throughout these sections we are going ask you to categorize your partners by their genitals, regardless of their gender. We understand that this is reductive, but we believe that this is the most scientifically relevant distinction. If you do not know your deep kissing partner's genitals, please classify the partner as you think is most appropriate.

Approximately how many deep kissing partners with a penis **have you had in the LAST SIX MONTHS?**

Number:

____

Q57 **Approximately how many deep kissing partners with** a penis **have you had in the LAST YEAR?**

Number:

____

Q58 **Approximately how many deep kissing partners** with a penis **have you had in your LIFETIME?**

Number:

____

End of Block: Kissing

Start of Block: Sexual Questions

X→

Q59
Personal Sexual Questions

The next set of questions is about your sexual behavior. By sex, we mean penetrative vaginal/front hole, oral, or anal sex. Penetrative sex includes (but is not necessarily limited to) penetration with a penis, finger, sex toy, or other object.

We understand that some questions may not have options that fully capture your experiences, but we ask that you select the answer that best fits. We are going to ask you very detailed questions about your sexual behavior and history. Reconstructing sexual histories will help us better understand human papillomavirus (HPV) infection risk.
Have you had any penetrative vaginal/front hole sex, oral sex, or anal sex sexual partners?

○ Yes (1)

○ No (0)

Skip To: End of Block If Personal Sexual Questions The next set of questions is about your sexual behavior. By sex, we m... != Yes

Page Break

Q60
A sexual partner is defined as a person with whom you engage in penetrative vaginal/front hole sex, oral sex, or anal sex. Penetrative sex includes (but is not necessarily limited to) penetration with a penis, finger, sex toy, or other object.

Thinking about when you have sex with a new partner for the first time, how often do you know whether or not they have a sexually transmitted infection (STI) before you have sex with them?

- ○ I always know whether or not my partners have STIs (1)
- ○ I sometimes know when my partners have STIs (2)
- ○ I never know whether or not my partners have STIs (3)
- ○ I have never had any sexual partners (4)

Q61 **How many of your sexual partners do you think have met each other?**

- ○ All (1)
- ○ Most (2)
- ○ Some (3)
- ○ None (4)
- ○ Don't know (888)

Q62 **How many of your sexual partners do you think have had sex with each other?**

- ○ All (1)
- ○ Most (2)
- ○ Some (3)
- ○ None (4)
- ○ Don't know (888)

Page Break

Q63 **Personal Sexual Questions**

The next set of questions is about your sexual behavior. By sex, we mean penetrative vaginal/front hole, oral, or anal sex. Penetrative sex includes (but is not necessarily limited to) penetration with a penis, finger, sex toy, or other object.

Because transmission of the human papillomavirus may depend on one's genitals, throughout these sections we are going ask you to categorize your partners by their genitals, regardless of their gender. We understand that this is reductive, but we believe that this is the most scientifically relevant distinction. If you do not know your partner's genitals, please classify the partner as you think is most appropriate.

Q64 **Approximately how many sexual partners with a vagina/front hole have you had in the LAST SIX MONTHS?**

Number:

____

Q65 **Approximately how many sexual partners with a vagina/front hole have you had in the LAST YEAR?**

Number:

____

Q66 **Approximately how many sexual partners with** a vagina/front hole **have you had in your LIFETIME?**

Number:

____

Page Break

Display This Question:

If If Approximately how many sexual partners with a vagina/front hole have you had in your LIFETIME? ... Text Response Is Greater Than 0

JS

Q67
Personal Sexual Questions

A sexual partner is defined as a person with whom you engage in penetrative vaginal/front hole sex, oral sex, or anal sex. Penetrative sex includes (but is not necessarily limited to) penetration with a penis, finger, sex toy, or other object.

Please indicate how many sexual partners with a vagina/front hole you had during each age range throughout your lifetime.

A new partner is defined as someone you had sex with for the first time during that age range.

A continuing partner is defined as someone you have had sex with before and listed as a "new partner" during any previous age range.

Display This Choice:
If Age >= 19
Display This Choice:
If Age >= 23
Display This Choice:
If Age >= 26
Display This Choice:
If Age >= 30
Display This Choice:
If Age >= 35
Display This Choice:
If Age >= 40
Display This Choice:
If Age >= 50
Display This Choice:
If Age >= 60

	Number of new partners with a vagina/front hole (1)	Number of continuing partners with a vagina/front hole (2)

Age (1)		
Age 16-18 (2)		
Display This Choice:<br>*If Age >= 19*<br>Age 19-22 (3)		
Display This Choice:<br>*If Age >= 23*<br>Age 23-25 (8)		
Display This Choice:<br>*If Age >= 26*<br>Age 26-29 (4)		
Display This Choice:<br>*If Age >= 30*<br>Age 30-34 (5)		
Display This Choice:<br>*If Age >= 35*<br>Age 35-39 (6)		

Display This Choice: If Age >= 40 Age 40-49 (7)		
Display This Choice: If Age >= 50 Age 50-59 (14)		
Display This Choice: If Age >= 60 Age 60+ (17)		

Page Break

Q68
A sexual partner is defined as a person with whom you engage in penetrative vaginal/front hole sex, oral sex, or anal sex. By penis, we mean a natal (by birth) or surgically constructed penis.
Approximately how many sexual partners with a penis have you had in the LAST SIX MONTHS?

Number:

____

Q69 **Approximately how many sexual partners with a penis have you had in the LAST YEAR?**

Number:

____

Q70 **Approximately how many sexual partners with a penis have you had in your LIFETIME?**

Number:

____

Display This Question:

If If Approximately how many sexual partners with a penis have you had in your LIFETIME?   Number: Text Response Is Greater Than 0

Q71

Have you ever had sexual partner who had an uncircumcised penis?
Select "no" for a surgically constructed penis.

- ○ Yes (1)
- ○ No (0)
- ○ Don't know (2)

Page Break

Display This Question:

If If Approximately how many sexual partners with a penis have you had in your LIFETIME?   Number: Text Response Is Greater Than 0

JS

Q72

Personal Sexual Questions

A sexual partner is defined as a person with whom you engage in penetrative vaginal/front hole sex, oral sex, or anal sex. By penis, we mean a natal (by birth) or surgically constructed penis.

Please indicate how many sexual partners with a penis you had during each age range throughout your lifetime.

A new partner is defined as someone you had sex with for the first time during that age range.

A continuing partner is defined as someone you have had sex with before and listed as a "new partner" during any previous age range.

Display This Choice:

If Age >= 19

Display This Choice:

If Age >= 23

Display This Choice:

If Age >= 26

Display This Choice:

If Age >= 30

Display This Choice:

If Age >= 35

Display This Choice:

If Age >= 40

Display This Choice:

If Age >= 50

Display This Choice:

If Age >= 60

	Number of new partners with a penis (1)	Number of continuing partners with a penis (2)

Age (1)		
Age 16-18 (2)		
Display This Choice:<br>*If Age >= 19*<br>Age 19-22 (3)		
Display This Choice:<br>*If Age >= 23*<br>Age 23-25 (8)		
Display This Choice:<br>*If Age >= 26*<br>Age 26-29 (4)		
Display This Choice:<br>*If Age >= 30*<br>Age 30-34 (5)		
Display This Choice:<br>*If Age >= 35*<br>Age 35-39 (6)		

Display This Choice: If Age >= 40 Age 40-49 (7)		
Display This Choice: If Age >= 50 Age 50-59 (14)		
Display This Choice: If Age >= 60 Age 60+ (17)		

Page Break

Q73

Personal Sexual Questions
Penetrative vaginal/front hole sex is defined as being involved in vaginal/front hole penetration with a partner, whether you are the giver or receiver. Penetrative vaginal/front hole sex includes (but is not necessarily limited to) penetration with a penis, finger, sex toy, or other object.

Have you ever had penetrative vaginal/front hole sex?

○ Yes (1)

○ No (0)

Skip To: Q87 If Personal Sexual Questions Penetrative vaginal/front hole sex is defined as being involved in va... != Yes

Page Break

Q74 **How old was your partner when you first had penetrative vaginal/front hole sex?**

Age:

____

Q75

Penetrative vaginal/front hole sex is defined as being involved in vaginal/front hole penetration with a partner, whether you are the giver or receiver. Penetrative vaginal/front hole sex includes (but is not necessarily limited to) penetration with a penis, finger, sex toy, or other object.

How old were you when you first had vaginal/front hole sex?

Age:

____

Q76 Which of the following best describes the gender and sex of your fir**st vaginal/front hole s**ex partner at the time?

- ○ Cisgender man (1)
- ○ Cisgender woman (2)
- ○ Transgender man (4)
- ○ Transgender woman (5)
- ○ Genderqueer/nonbinary/agender, assigned male at birth (6)
- ○ Genderqueer/nonbinary/agender, assigned female at birth (3)

Page Break

Q77
Penetrative vaginal/front hole sex is defined as being involved in vaginal/front hole penetration with a partner, whether you are the giver or receiver. Penetrative vaginal/front hole sex includes (but is not necessarily limited to) penetration with a penis, finger, sex toy, or other object.
How many vaginal/front hole sex partners with a vagina/front hole have you had in the LAST SIX MONTHS?

Number:

____

Q78 **How many vaginal/front hole sex partners with a vagina/front hole have you had in the LAST YEAR?**

Number:

____

Q79 **How many vaginal/front hole sex partners with a vagina/front hole have you had in your LIFETIME?**

Number:

____

Page Break

Display This Question:

If If How many vaginal/front hole sex partners with a vagina/front hole have you had in your LIFETIME?   Number: Text Response Is Greater Than 0

Q80

Personal Sexual Questions

Penetrative vaginal/front hole sex is defined as being involved in vaginal/front hole penetration with a partner, whether you are the giver or receiver. Penetrative vaginal/front hole sex includes (but is not necessarily limited to) penetration with a penis, finger, sex toy, or other object.

Please indicate how vaginal/front hole sexual partners with a vagina/front hole you had during each age range throughout your lifetime.

A new partner is defined as someone you had sex with for the first time during that age range.

A continuing partner is defined as someone you have had sex with before and listed as a "new partner" during any previous age range.

Display This Choice:

If Age >= 19

Display This Choice:

If Age >= 23

Display This Choice:

If Age >= 26

Display This Choice:

If Age >= 30

Display This Choice:

If Age >= 35

Display This Choice:

If Age >= 40

Display This Choice:

If Age >= 50

Display This Choice:

If Age >= 60

	Number of new partners with a vagina/front hole (1)	Number of continuing partners with a vagina/front hole (2)

Age (1)		
Age 16-18 (2)		
Display This Choice: If Age >= 19 Age 19-22 (3)		
Display This Choice: If Age >= 23 Age 23-25 (8)		
Display This Choice: If Age >= 26 Age 26-29 (4)		
Display This Choice: If Age >= 30 Age 30-34 (5)		
Display This Choice: If Age >= 35 Age 35-39 (6)		

Display This Choice: If Age >= 40 Age 40-49 (7)		
Display This Choice: If Age >= 50 Age 50-59 (14)		
Display This Choice: If Age >= 60 Age 60+ (17)		

Page Break

Display This Question:

If What sex were you assigned at birth (i.e., on your original birth certificate)? != Male

*

Q81

Personal Sexual Questions

Penetrative vaginal/front hole sex is defined as being involved in vaginal/front hole penetration with a partner, whether you are the giver or receiver. Penetrative vaginal/front hole sex includes (but is not necessarily limited to) penetration with a penis, finger, sex toy, or other object. By penis, we mean a natal (by birth) or surgically constructed penis.

How many vaginal/front hole sex partners with a penis have you had in the LAST SIX MONTHS?

Number:

____

Display This Question:

If What sex were you assigned at birth (i.e., on your original birth certificate)? != Male

*

Q82 **How many vaginal/front hole sex partners with a penis have you had in the LAST YEAR?**

Number:

____

Display This Question:

If What sex were you assigned at birth (i.e., on your original birth certificate)? != Male

*

Q83 **How many vaginal/front hole sex partners with a penis have you had in your LIFETIME?**

Number:

____

Page Break

Display This Question:

If If How many vaginal/front hole sex partners with a penis have you had in your LIFETIME? Number: Text Response Is Greater Than 0

JS

Q84

Personal Sexual Questions

Penetrative vaginal/front hole sex is defined as being involved in vaginal/front hole penetration with a partner, whether you are the giver or receiver. Penetrative vaginal/front hole sex includes (but is not necessarily limited to) penetration with a penis, finger, sex toy, or other object. By penis, we mean a natal (by birth) or surgically constructed penis.

Please indicate how many vaginal/front hole sexual partners with a penis you had during each age range throughout your lifetime.

A new partner is defined as someone you had sex with for the first time during that age range.

A continuing partner is defined as someone you have had sex with before and listed as a "new partner" during any previous age range.

Display This Choice:

If Age >= 19

Display This Choice:

If Age >= 23

Display This Choice:

If Age >= 26

Display This Choice:

If Age >= 30

Display This Choice:

If Age >= 35

Display This Choice:

If Age >= 40

Display This Choice:

If Age >= 50

Display This Choice:

If Age >= 60

	Number of new partners with a penis (1)	Number of continuing partners with a penis (2)

Age (1)		
Age 16-18 (2)		
Display This Choice:<br>*If Age >= 19*<br>Age 19-22 (3)		
Display This Choice:<br>*If Age >= 23*<br>Age 23-25 (8)		
Display This Choice:<br>*If Age >= 26*<br>Age 26-29 (4)		
Display This Choice:<br>*If Age >= 30*<br>Age 30-34 (5)		
Display This Choice:<br>*If Age >= 35*<br>Age 35-39 (6)		

Display This Choice: *If Age >= 40* Age 40-49 (7)		
Display This Choice: *If Age >= 50* Age 50-59 (14)		
Display This Choice: *If Age >= 60* Age 60+ (17)		

Page Break

Q85

Personal Sexual Questions
Penetrative vaginal/front hole sex is defined as being involved in vaginal/front hole penetration with a partner, whether you are the giver or receiver. Penetrative vaginal/front hole sex includes (but is not necessarily limited to) penetration with a penis, finger, sex toy, or other object.
How often do you use barriers such as condoms or finger cots during vaginal/front hole sex?

- Always (1)
- Most of the time (2)
- Sometimes (3)
- Rarely (4)
- Never (5)
- Don't know (888)

Display This Question:

If Personal Sexual Questions Penetrative vaginal/front hole sex is defined as being involved in va... != Never

Q86 **What kind of barriers do you use?**

- Condoms (1)
- Finger cots (2)
- Other: (3) ____

Page Break

Q87

Personal Sexual Questions
Oral sex is defined as the stimulation of genitalia using the mouth or throat.

Have you ever had oral sex (as either the giver or receiver)?

- Yes (1)
- No (0)

Skip To: Q113 If Personal Sexual Questions Oral sex is defined as the stimulation of genitalia using the mouth o... != Yes

Page Break

Q88

Personal Sexual Questions
Oral sex is defined as the stimulation of genitalia using the mouth or throat.

How old were you when you first had oral sex?

Age:

____

Q89 **How old was your partner when you first had oral sex?**

Age:

____

Q90 Which of the following best describes the gender and sex of your first oral sex partner at the time?

- ◯ Cisgender man (1)
- ◯ Cisgender woman (2)
- ◯ Transgender man (4)
- ◯ Transgender woman (5)
- ◯ Genderqueer/nonbinary/agender, assigned male at birth (6)
- ◯ Genderqueer/nonbinary/agender, assigned female at birth (3)

Page Break

Q91 Oral sex is defined as the stimulation of genitalia using the mouth or throat. Because transmission of the human papillomavirus may depend on one's genitals, throughout these sections we are going ask you to categorize your partners by their genitals, regardless of their gender. We understand that this is reductive, but we believe that this is the most scientifically relevant distinction. If you do not know your partner's genitals, please classify the partner as you think is most appropriate.

In some questions, we are going to ask you to distinguish between performing and receiving oral sex.

How many oral sex partners with a vagina/front hole have you had in the LAST SIX MONTHS?

Number:

____

Display This Question:

If If Oral sex is defined as the stimulation of genitalia using the mouth or throat. Because transmission of the human papillomavirus may depend on one's genitals, throughout these sections we are ... Text Response Is Greater Than 0

Q92 **In the LAST SIX MONTHS, on how many partners with a vagina/front hole have you PERFORMED oral sex?**

Number:

____

Display This Question:

If If Oral sex is defined as the stimulation of genitalia using the mouth or throat. Because transmission of the human papillomavirus may depend on one's genitals, throughout these sections we are ... Text Response Is Greater Than 0

Q93 **In the LAST SIX MONTHS, from how many partners with a vagina/front hole have you RECEIVED oral sex?**

Number:

____

Q94 **How many oral sex partners with a vagina/front hole have you had in the LAST YEAR?**

Number:

____

Display This Question:

If If How many oral sex partners with a vagina/front hole have you had in the LAST YEAR?   Number: Text Response Is Greater Than 0

Q95 **In the LAST YEAR, on how many partners with a vagina/front hole have you PERFORMED oral sex?**

Number:

____

Display This Question:

If If Oral sex is defined as the stimulation of genitalia using the mouth or throat. Because transmission of the human papillomavirus may depend on one's genitals, throughout these sections we are ... Text Response Is Greater Than 0

Q96 **In the LAST YEAR, from how many partners with a vagina/front hole have you RECEIVED oral sex?**

Number:

____

Q97 **How many oral sex partners with a vagina/front hole have you had in your LIFETIME?**

Number:

____

Display This Question:

If If How many oral sex partners with a vagina/front hole have you had in your LIFETIME?   Number: Text Response Is Greater Than 0

Q98 **In your LIFETIME, on how many partners with a vagina/front hole have you PERFORMED oral sex?**

Number:

____

Display This Question:

If If How many oral sex partners with a vagina/front hole have you had in your LIFETIME?   Number: Text Response Is Greater Than 0

Q99 **In your LIFETIME, from how many partners with a vagina/front hole have you RECEIVED oral sex?**

Number:

____

Page Break

Display This Question:

If If In your LIFETIME, on how many partners with a vagina/front hole have you PERFORMED oral sex?   Number: Text Response Is Greater Than 0

Or Or In your LIFETIME, from how many partners with a vagina/front hole have you RECEIVED oral sex?   Number: Text Response Is Greater Than 0

JS

Q100

Personal Sexual Questions

Oral sex is defined as the stimulation of genitalia using the mouth or throat.

Please indicate how many oral sexual partners (giving or receiving) with a vagina/front hole you had during each age range throughout your lifetime.

A new partner is defined as someone you had sex with for the first time during that age range.

A continuing partner is defined as someone you have had sex with before and listed as a "new partner" during any previous age range.

Display This Choice:

If Age >= 19

Display This Choice:

If Age >= 23

Display This Choice:

If Age >= 26

Display This Choice:

If Age >= 30

Display This Choice:

If Age >= 35

Display This Choice:

If Age >= 40

Display This Choice:

If Age >= 50

Display This Choice:

If Age >= 60

	Number of new partners with a vagina/front hole (1)	Number of continuing partners with a vagina/front hole (2)

Age (1)		
Age 16-18 (2)		
Display This Choice: If Age >= 19 Age 19-22 (3)		
Display This Choice: If Age >= 23 Age 23-25 (8)		
Display This Choice: If Age >= 26 Age 26-29 (4)		
Display This Choice: If Age >= 30 Age 30-34 (5)		
Display This Choice: If Age >= 35 Age 35-39 (6)		

Display This Choice: *If Age >= 40* Age 40-49 (7)		
Display This Choice: *If Age >= 50* Age 50-59 (14)		
Display This Choice: *If Age >= 60* Age 60+ (17)		

Page Break

Q101

Personal Sexual Questions
Oral sex is defined as the stimulation of genitalia using the mouth or throat. By penis, we mean a natal (by birth) or surgically constructed penis.

How many assigned oral sex partners with a penis have you had in the <u>LAST SIX MONTHS</u>?

Number:

Display This Question:

If If Personal Sexual Questions   Oral sex is defined as the stimulation of genitalia using the mouth or throat. By penis, we mean a natal (by birth) or surgically constructed penis.   ... Text Response Is Greater Than 0

Q102 **In the <u>LAST SIX MONTHS</u>, on how many partners with a penis have you <u>PERFORMED</u> oral sex?**

Number:

Display This Question:

If If Personal Sexual Questions   Oral sex is defined as the stimulation of genitalia using the mouth or throat. By penis, we mean a natal (by birth) or surgically constructed penis.   ... Text Response Is Greater Than 0

Q103
In the <u>LAST SIX MONTHS</u>, from how many partners with a penis have you <u>RECEIVED</u> oral sex?

Number:

____

Q104 **How many oral sex partners with a penis have you had in the LAST YEAR?**

Number:

____

Display This Question:

If If How many oral sex partners with a penis have you had in the LAST YEAR?   Number: Text Response Is Greater Than 0

Q105 **In the LAST YEAR, on how many partners with a penis have you PERFORMED oral sex?**

Number:

____

Display This Question:

If If How many oral sex partners with a penis have you had in the LAST YEAR?   Number: Text Response Is Greater Than 0

Q106 **In the LAST YEAR, from how many partners with a penis have you RECEIVED oral sex?**

Number:

____

Q107 **How many oral sex partners with a penis have you had in your <u>LIFETIME</u>?**

Number:

____

Display This Question:

If If How many oral sex partners with a penis have you had in your LIFETIME?   Number: Text Response Is Greater Than 0

Q108 **In your <u>LIFETIME</u>, on how many partners with a penis have you <u>PERFORMED</u> oral sex?**

Number:

____

Display This Question:

If If How many oral sex partners with a penis have you had in your LIFETIME?   Number: Text Response Is Greater Than 0

Q109 **In your <u>LIFETIME</u>, from how many partners with a penis have you <u>RECEIVED</u> oral sex?**

Number:

____

Page Break

Display This Question:

If If In your LIFETIME, on how many partners with a penis have you PERFORMED oral sex?   Number: Text Response Is Greater Than 0

Or Or In your LIFETIME, from how many partners with a penis have you RECEIVED oral sex?   Number: Text Response Is Greater Than 0

JS

Q110

Personal Sexual Questions
Oral sex is defined as the stimulation of genitalia using the mouth or throat. By penis, we mean a natal (by birth) or surgically constructed penis.

Please indicate how many sexual partners (giving or receiving) with a penis you had during each age range throughout your lifetime.

A new partner is defined as someone you had sex with for the first time during that age range.

A continuing partner is defined as someone you have had sex with before and listed as a "new partner" during any previous age range.

Display This Choice:

If Age >= 19

Display This Choice:

If Age >= 23

Display This Choice:

If Age >= 26

Display This Choice:

If Age >= 30

Display This Choice:

If Age >= 35

Display This Choice:

If Age >= 40

Display This Choice:

If Age >= 50

Display This Choice:

If Age >= 60

	Number of new partners with a penis (1)	Number of continuing partners with a penis (2)

Age (1)		
Age 16-18 (2)		
Display This Choice: If Age >= 19 Age 19-22 (3)		
Display This Choice: If Age >= 23 Age 23-25 (8)		
Display This Choice: If Age >= 26 Age 26-29 (4)		
Display This Choice: If Age >= 30 Age 30-34 (5)		
Display This Choice: If Age >= 35 Age 35-39 (6)		

Display This Choice:<br>*If Age >= 40*<br>Age 40-49 (7)		
Display This Choice:<br>*If Age >= 50*<br>Age 50-59 (14)		
Display This Choice:<br>*If Age >= 60*<br>Age 60+ (17)		

Page Break

Q111

Personal Sexual Questions
Oral sex is defined as the stimulation of genitalia using the mouth or throat.

How often do you use barriers such as condoms or dams during oral sex?

- Always (1)
- Most of the time (2)
- Sometimes (3)
- Rarely (4)
- Never (5)
- Don't know (888)

Display This Question:

If Personal Sexual Questions Oral sex is defined as the stimulation of genitalia using the mouth o... != Never

Q112 **What kind of barriers do you use?**

- Dental dams or plastic wrap (1)
- Condoms (2)
- Other: (3) ____

Page Break

Q113

Personal Sexual Questions
Penetrative anal sex is defined as anal penetration (giving or receiving) with a partner. Penetrative anal sex includes (but is not necessarily limited to) penetration with a penis, finger, sex toy, or other object.

Have you ever had penetrative anal sex?

- Yes (1)
- No (0)

Skip To: End of Block If Personal Sexual Questions Penetrative anal sex is defined as anal penetration (giving or receiv... != Yes

Page Break

Q114 **How old were you when you first had penetrative anal sex?**

Age:

____

Q115 **How old was your partner when you first had penetrative anal sex?**

Age:

____

Q116 Which of the following best describes the gender and sex of your first anal sex partner at the time?

- ○ Cisgender man (1)
- ○ Cisgender woman (2)
- ○ Transgender man (4)
- ○ Transgender woman (5)
- ○ Genderqueer/nonbinary/agender, assigned male at birth (6)
- ○ Genderqueer/nonbinary/agender, assigned female at birth (3)

Page Break

*

Q117
Penetrative anal sex is defined as anal penetration (giving or receiving) with a partner. Penetrative anal sex includes (but is not necessarily limited to) penetration with a penis, finger, sex toy, or other object. Because transmission of the human papillomavirus may depend on one's genitals, throughout these sections we are going ask you to categorize your partners by their genitals, regardless of their gender. We understand that this is reductive, but we believe that this is the most scientifically relevant distinction. If you do not know your partner's genitals, please classify the partner as you think is most appropriate.
How many penetrative anal sex partners with a vagina/front hole have you had in the LAST SIX MONTHS?

Number:

____

*

Q118 **How many anal sex partners with a vagina/front hole have you had in the LAST YEAR?**

Number:

____

*

Q119 **How many penetrative anal sex partners with a vagina/front hole have you had in your LIFETIME?**

Number:

____

Page Break

Display This Question:

If If How many penetrative anal sex partners with a vagina/front hole have you had in your LIFETIME?   Number: Text Response Is Greater Than 0

Q120

Personal Sexual Questions

Penetrative anal sex is defined as anal penetration (giving or receiving) with a partner. Penetrative anal sex includes (but is not necessarily limited to) penetration with a penis, finger, sex toy, or other object.

Please indicate how many penetrative anal sexual partners with a vagina/front hole you had during each age range throughout your lifetime.

A new partner is defined as someone you had sex with for the first time during that age range.

A continuing partner is defined as someone you have had sex with before and listed as a "new partner" during any previous age range.

Display This Choice:
If Age >= 19
Display This Choice:
If Age >= 23
Display This Choice:
If Age >= 26
Display This Choice:
If Age >= 30
Display This Choice:
If Age >= 35
Display This Choice:
If Age >= 40
Display This Choice:
If Age >= 50
Display This Choice:
If Age >= 60

	Number of new partners with a vagina/front hole (1)	Number of continuing partners with a vagina/front hole (2)

Age (1)		
Age 16-18 (2)		
Display This Choice: If Age >= 19 Age 19-22 (3)		
Display This Choice: If Age >= 23 Age 23-25 (8)		
Display This Choice: If Age >= 26 Age 26-29 (4)		
Display This Choice: If Age >= 30 Age 30-34 (5)		
Display This Choice: If Age >= 35 Age 35-39 (6)		

Display This Choice:<br>*If Age >= 40*<br>Age 40-49 (7)		
Display This Choice:<br>*If Age >= 50*<br>Age 50-59 (14)		
Display This Choice:<br>*If Age >= 60*<br>Age 60+ (17)		

Page Break

Q121
Penetrative anal sex is defined as anal penetration (giving or receiving) with a partner. Penetrative anal sex includes (but is not necessarily limited to) penetration with a penis, finger, sex toy, or other object. By penis, we mean a natal (by birth) or surgically constructed penis.

How many penetrative anal sex partners with a penis have you had in the LAST SIX MONTHS?

Number:

____

Q122 **How many penetrative anal sex partners with a penis have you had in the LAST YEAR?**

Number:

____

Q123 **How many penetrative anal sex partners with a penis have you had in your LIFETIME?**

Number:

____

Page Break

Display This Question:

If If In your LIFETIME, on how many partners with a penis have you PERFORMED oral sex?   Number: Text Response Is Greater Than 0

JS

Q124

Personal Sexual Questions

Penetrative anal sex is defined as anal penetration (giving or receiving) with a partner. Penetrative anal sex includes (but is not necessarily limited to) penetration with a penis, finger, sex toy, or other object. By penis, we mean a natal (by birth) or surgically constructed penis.

Please indicate how many penetrative anal sexual partners with a penis you had during each age range throughout your lifetime.

A new partner is defined as someone you had sex with for the first time during that age range.

A continuing partner is defined as someone you have had sex with before and listed as a "new partner" during any previous age range.

Display This Choice:

If Age >= 19

Display This Choice:

If Age >= 23

Display This Choice:

If Age >= 26

Display This Choice:

If Age >= 30

Display This Choice:

If Age >= 35

Display This Choice:

If Age >= 40

Display This Choice:

If Age >= 50

Display This Choice:

If Age >= 60

	Number of new partners with a penis (1)	Number of continuing partners with a penis (2)

Age (1)		
Age 16-18 (2)		
Display This Choice: If Age >= 19 Age 19-22 (3)		
Display This Choice: If Age >= 23 Age 23-25 (8)		
Display This Choice: If Age >= 26 Age 26-29 (4)		
Display This Choice: If Age >= 30 Age 30-34 (5)		
Display This Choice: If Age >= 35 Age 35-39 (6)		

Display This Choice:<br>*If Age >= 40*<br>Age 40-49 (7)		
Display This Choice:<br>*If Age >= 50*<br>Age 50-59 (14)		
Display This Choice:<br>*If Age >= 60*<br>Age 60+ (17)		

Page Break

Q125 **When having penetrative anal sex, how often are you the giver versus the receiver?**

- I am always the giver and never the receiver. (1)
- I am the giver most of the time, but have been the receiver at least once. (2)
- I give and receive anal sex about equally. (3)
- I am the receiver most of the time, but have been the giver at least once. (4)
- I am always the receiver and never the giver. (5)
- Don't know (888)

Q126 **How often do you use barriers like condoms or finder cots during penetrative anal sex?**

- Always (1)
- Most of the time (2)
- Sometimes (3)
- Rarely (4)
- Never (5)
- Don't know (888)

Display This Question:

If How often do you use barriers like condoms or finder cots during penetrative anal sex? != Never

Q127 **What kind of barriers do you use?**

- ☐ Condoms (1)
- ☐ Finger cots (2)
- ☐ Other: (3) ____

Page Break

End of Block: Sexual Questions

Start of Block: Genital Touching

Q128

Personal Sexual Questions
Non-penetrative sex may involve self-stimulation or your stimulation of your partner and may include certain kinds of genital touching or mutual masturbation.

Have you ever practiced non-penetrative sex with another person?

○ Yes (1)

○ No (0)

Skip To: End of Block If Personal Sexual Questions Non-penetrative sex may involve self-stimulation or your stimulation... != Yes

Page Break

Q129

Personal Sexual Questions
Non-penetrative sex includes certain kinds of genital touching and mutual masturbation, often with hands, that may involve self-stimulation or your stimulation of your partner.

How old were you when you first practiced non-penetrative sex with a partner?

Age:

____

Q130 **How old was your partner when you first practiced** non-penetrative sex**?**

Age:

____

End of Block: Genital Touching

Start of Block: Rimming

Q131

Personal Sexual Questions
Rimming, anilingus, or oral-anal contact involves stimulating or contacting the anus with your mouth, tongue, or teeth.

Have you ever practiced rimming or oral-anal contact with another person?

○ Yes (1)

○ No (0)

Skip To: End of Block If Personal Sexual Questions Rimming, anilingus, or oral-anal contact involves stimulating or cont... != Yes

Page Break

Q132

Personal Sexual Questions
Rimming, anilingus, or oral-anal contact involves stimulating or contacting the anus with your mouth, tongue, or teeth.

How old were you when you first practiced rimming or oral-anal contact?

Age:

____

Q133 **How old was your partner when you first practiced rimming or oral-anal contact?**

Age:

____

End of Block: Rimming

Start of Block: Number of Recent Partners

Q134

Personal Sexual Questions

In the previous sections, you told us about the numbers of sexual partners you have had. We are now going to ask you more detailed questions about your three most recent partners in the last six months. In order to personalize the next section for you, please re-enter the number of sexual partners you have had in the last six months. A sexual partner is defined as a person with whom you engage in penetrative vaginal/front hole sex, oral sex, or anal sex. Penetrative sex includes (but is not necessarily limited to) penetration with a penis, finger, sex toy, or other object.
How many sexual partners have you had in the last six months?

____

End of Block: Number of Recent Partners

Start of Block: Most Recent Partner

Display This Question:

If If Personal Sexual Questions In the previous sections, you told us about the numbers of sexual partners you have had. We are now going to ask you more detailed questions about your three most recen... Text Response Is Greater Than 0

X→

Q135
The following questions are about your MOST RECENT partner.

What was the gender and sex of this partner?

- ◯ Cisgender man (1)
- ◯ Cisgender woman (2)
- ◯ Transgender man (4)
- ◯ Transgender woman (5)
- ◯ Genderqueer/nonbinary/agender, assigned male at birth (6)
- ◯ Genderqueer/nonbinary/agender, assigned female at birth (3)

Page Break

Q136 **How would your describe your relationship with this partner?**

- ○ Committed, monogamous romantic relationship (1)
- ○ Committed, non-monogamous romantic relationship (2)
- ○ Uncommitted, romantic relationship (e.g., dating) (3)
- ○ Non-romantic relationship (e.g., "friends with benefits") (4)
- ○ Single encounter (e.g., "one night stand") (5)
- ○ Other (6) ____

Q137 **Did this partner have a penis, and were they circumcised?**
Circumcision status can affect HPV transmission.

- ○ Partner did not have a penis (1)
- ○ Partner had a penis and was circumcised (2)
- ○ Partner had a penis and was uncircumcised (3)
- ○ Partner had a surgically constructed penis (4)
- ○ Don't know (888)

Q138 **What sexual acts do/did you typically engage in with this partner (check all that apply)?**

- ☐ Vaginal/front hole receiving (1)
- ☐ Vaginal/front hole giving (2)
- ☐ Oral receiving (3)
- ☐ Oral giving (4)
- ☐ Anal receiving (5)
- ☐ Anal giving (6)
- ☐ Rimming receiving (7)
- ☐ Rimming giving (8)
- ☐ Sex toys (9)
- ☐ Non-penetrative sex receiving (10)
- ☐ Non-penetrative sex giving (11)
- ☐ Other: (12) ____
- ☐ ⊗Prefer not to answer (999)

---

*

Q139 **During the last year, how often did you have sex with this partner (# times/year)?**

____

---

*

Q140 **What was your partner’s age at the time of your last sexual encounter with them?**
If you are unsure, give your best guess.

____

Q141 **What was your age the <u>first time</u> you had sex with this partner?**

____

Q142 **What was your age the <u>last time</u> you had sex with this partner?**

____

Q143 **Did you ever use your saliva as lubricant during penetrative sex with this partner?**

- ◯ Yes (1)
- ◯ No (0)

Q144 **Did you ever use your partner's saliva as lubricant during penetrative sex?**

- ◯ Yes (1)
- ◯ No (0)

Q145 **Did you ever use lubricant (other than saliva) when having penetrative sex with this partner?**

- Yes (1)
- No (0)

Q146 **How often did you use a condom or similar protection during your sexual encounters with this partner?**

- Always (1)
- Most of the time (2)
- Sometimes (3)
- Rarely (4)
- Never (5)
- Don't know (888)

Q147 **The last time you had sex with this partner, did you use a condom or similar protection?**

- Yes (1)
- No (0)

Q148 **Do you expect to have sex with this partner again?**

- ○ Yes (1)
- ○ No (0)
- ○ Don't know (999)

X→

Q149
While this person was your sexual partner, what was their HIV (human immunodeficiency virus) status?

- ○ This partner was never tested for HIV (1)
- ○ This partner tested negative for HIV (2)
- ○ This partner tested positive for HIV (3)
- ○ Don't know (888)

X→

Q150 **While this person was your sexual partner, what was their HPV (human papillomavirus) status?**

- ○ This partner was never tested for HPV (1)
- ○ This partner tested negative for HPV (2)
- ○ This partner tested positive for HPV (3)
- ○ Don't know (888)

X→

Q151
While this person was your sexual partner, what was their STI status other than HIV and HPV?

- ◯ This partner was never tested for other STIs (1)
- ◯ This partner tested negative for all other STIs (2)
- ◯ This partner tested positive for at least one other STI (please enter below): (3)
____
- ◯ Don't know (888)

X→

Q152 **During any of your sexual encounters with this partner, did you have an STI that you were aware of? If so, which one?**

- ◯ Yes (1) ____
- ◯ No (0)

End of Block: Most Recent Partner

Start of Block: Second Most Recent Partner

Display This Question:

If If Personal Sexual Questions In the previous sections, you told us about the numbers of sexual partners you have had. We are now going to ask you more detailed questions about your three most recen... Text Response Is Greater Than 1

X→

Q153
The following questions are about your SECOND MOST RECENT partner.

What was the gender and sex of this partner?

- ○ Cisgender man (1)
- ○ Cisgender woman (2)
- ○ Transgender man (4)
- ○ Transgender woman (5)
- ○ Genderqueer/nonbinary/agender, assigned male at birth (6)
- ○ Genderqueer/nonbinary/agender, assigned female at birth (3)

Page Break

Q154 How would your describe your relationship with this partner?

- ○ Committed, monogamous romantic relationship (1)
- ○ Committed, non-monogamous romantic relationship (2)
- ○ Uncommitted, romantic relationship (e.g., dating) (3)
- ○ Non-romantic relationship (e.g., "friends with benefits") (4)
- ○ Single encounter (e.g., "one night stand") (5)
- ○ Other (6) ____

Q155 **Did this partner have a penis and were they circumcised?**
Circumcision status can affect HPV transmission.

- ○ Partner did not have a penis (1)
- ○ Partner had a penis and was circumcised (2)
- ○ Partner had a penis and was uncircumcised (3)
- ○ Partner had a surgically constructed penis (4)
- ○ Don't know (888)

Q156 What sexual acts do/did you typically engage in with this partner (check all that apply)?

- ☐ Vaginal/front hole receiving (1)
- ☐ Vaginal/front hole giving (2)
- ☐ Oral receiving (3)
- ☐ Oral giving (4)
- ☐ Anal receiving (5)
- ☐ Anal giving (6)
- ☐ Rimming receiving (7)
- ☐ Rimming giving (8)
- ☐ Sex toys (9)
- ☐ Non-penetrative sex receiving (10)
- ☐ Non-penetrative sex giving (11)
- ☐ Other: (12) ____
- ☐ ⊗Prefer not to answer (999)

---

*

Q157 **During the <u>last year</u>, how often did you have sex with this partner (# times/year)?**

____

---

*

Q158 **What was your partner’s age at the time of your last sexual encounter with them?**
If you are unsure, give your best guess.

____

Q159 **What was your age the <u>first time</u> you had sex with this partner?**

____

Q160 **What was your age the <u>last time</u> you had sex with this partner?**

____

Q161 **Did you ever use your saliva as lubricant during penetrative sex with this partner?**

- ○ Yes (1)
- ○ No (0)

Q162 **Did you ever use your partner's saliva as lubricant during penetrative sex?**

- ○ Yes (1)
- ○ No (0)

Q163 **Did you ever use lubricant (other than saliva) when having penetrative sex with this partner?**

- Yes (1)
- No (0)

Q164 **How often did you use a condom or similar protection during your sexual encounters with this partner?**

- Always (1)
- Most of the time (2)
- Sometimes (3)
- Rarely (4)
- Never (5)
- Don't know (888)

Q165 **The last time you had sex with this partner, did you use a condom or similar protection?**

- Yes (1)
- No (0)

Q166 **Do you expect to have sex with this partner again?**

- Yes (1)
- No (0)
- Don't know (999)

Q167
While this person was your sexual partner, what was their HIV (human immunodeficiency virus) status?

- This partner was never tested for HIV (1)
- This partner tested negative for HIV (2)
- This partner tested positive for HIV (3)
- Don't know (888)

Q168 **While this person was your sexual partner, what was their HPV (human papillomavirus) status?**

- This partner was never tested for HPV (1)
- This partner tested negative for HPV (2)
- This partner tested positive for HPV (3)
- Don't know (888)

Q169
While this person was your sexual partner, what was their STI status other than HIV and HPV?

○ This partner was never tested for other STIs (1)

○ This partner tested negative for all other STIs (2)

○ This partner tested positive for at least one other STI (please enter below): (3)
____

○ Don't know (888)

X→

Q170 **During any of your sexual encounters with this partner, did you have an STI that you were aware of? If so, which one?**

○ Yes (1) ____

○ No (0)

End of Block: Second Most Recent Partner

Start of Block: Third Most Recent Partner

Display This Question:

If If Personal Sexual Questions In the previous sections, you told us about the numbers of sexual partners you have had. We are now going to ask you more detailed questions about your three most recen... Text Response Is Greater Than 2

X→

Q171
The following questions are about your THIRD MOST RECENT partner.

What was the gender and sex of this partner?

- ○ Cisgender man (1)
- ○ Cisgender woman (2)
- ○ Transgender man (4)
- ○ Transgender woman (5)
- ○ Genderqueer/nonbinary/agender, assigned male at birth (3)
- ○ Genderqueer/nonbinary/agender, assigned female at birth (6)

Page Break

Q172 How would your describe your relationship with this partner?

- ○ Committed, monogamous romantic relationship (1)
- ○ Committed, non-monogamous romantic relationship (2)
- ○ Uncommitted, romantic relationship (e.g., dating) (3)
- ○ Non-romantic relationship (e.g., "friends with benefits") (4)
- ○ Single encounter (e.g., "one night stand") (5)
- ○ Other (6) ____

Q173 **Did this partner have a penis and were they circumcised?**
Circumcision status can affect HPV transmission.

- ○ Partner did not have a penis (1)
- ○ Partner had a penis and was circumcised (2)
- ○ Partner had a penis and was uncircumcised (3)
- ○ Partner had a surgically constructed penis (4)
- ○ Don't know (888)

Q174 What sexual acts do/did you typically engage in with this partner (check all that apply)?

- ▢ Vaginal/front hole receiving (1)
- ▢ Vaginal/front hole giving (2)
- ▢ Oral receiving (3)
- ▢ Oral giving (4)
- ▢ Anal receiving (5)
- ▢ Anal giving (6)
- ▢ Rimming receiving (7)
- ▢ Rimming giving (8)
- ▢ Sex toys (9)
- ▢ Non-penetrative sex receiving (10)
- ▢ Non-penetrative sex giving (11)
- ▢ Other: (12) ____
- ▢ ⊗Prefer not to answer (999)

---

*

Q175 **During the __last year__, how often did you have sex with this partner (# times/year)?**

____

---

*

Q176 **What was your partner’s age at the time of your last sexual encounter with them?**
If you are unsure, give your best guess.

____

Q177 **What was your age the <u>first time</u> you had sex with this partner?**

____

Q178 **What was your age the <u>last time</u> you had sex with this partner?**

____

Q179 **Did you ever use your saliva as lubricant during penetrative sex with this partner?**

- Yes (1)
- No (0)

Q180 **Did you ever use your partner's saliva as lubricant during penetrative sex?**

- Yes (1)
- No (0)

Q181 **Did you ever use lubricant (other than saliva) when having penetrative sex with this partner?**

- ◯ Yes (1)
- ◯ No (0)

Q182 **How often did you use a condom or similar protection during your sexual encounters with this partner?**

- ◯ Always (1)
- ◯ Most of the time (2)
- ◯ Sometimes (3)
- ◯ Rarely (4)
- ◯ Never (5)
- ◯ Don't know (888)

Q183 **The last time you had sex with this partner, did you use a condom or similar protection?**

- ◯ Yes (1)
- ◯ No (0)

Q184 **Do you expect to have sex with this partner again?**

- ○ Yes (1)
- ○ No (0)
- ○ Don't know (999)

Q185
While this person was your sexual partner, what was their HIV (human immunodeficiency virus) status?

- ○ This partner was never tested for HIV (1)
- ○ This partner tested negative for HIV (2)
- ○ This partner tested positive for HIV (3)
- ○ Don't know (888)

Q186 **While this person was your sexual partner, what was their HPV (human papillomavirus) status?**

- ○ This partner was never tested for HPV (1)
- ○ This partner tested negative for HPV (2)
- ○ This partner tested positive for HPV (3)
- ○ Don't know (888)

Q187
While this person was your sexual partner, what was their STI status other than HIV and HPV?

○ This partner was never tested for other STIs (1)

○ This partner tested negative for all other STIs (2)

○ This partner tested positive for at least one other STI (please enter below): (3)
____

○ Don't know (888)

Q188 **During any of your sexual encounters with this partner, did you have an STI that you were aware of? If so, which one?**

○ Yes (1) ____

○ No (0)

End of Block: Third Most Recent Partner

Start of Block: Substance Use

Q189
Substance Use

The following questions are about your substance use/abuse. We are asking because substance use/abuse may be correlated with human papillomavirus risk (HPV).

Have you ever smoked cigarettes regularly?

- ○ Yes, and still do (1)
- ○ Yes, but not currently (2)
- ○ No (0)

Skip To: Q195 If Substance Use The following questions are about your substance use/abuse. We are asking because... != Yes, and still do

Page Break

Q190 **At what age did you start smoking cigarettes regularly?**

Age:

Q191 **At each age range: average number of cigarettes per day**

Display This Choice:

If Age >= 19

Display This Choice:

If Age >= 26

Display This Choice:

If Age >= 30

Display This Choice:

If Age >= 40

Display This Choice:

If Age >= 50

Display This Choice:

If Age >= 60

	None (1)	1-4 (2)	5-14 (3)	15-24 (4)	25-34 (5)	35-44 (6)	45+ (7)	Prefer not to answer (999)
Age (Q254_1)	○	○	○	○	○	○	○	○
Age 16-18 (Q254_2)	○	○	○	○	○	○	○	○
Display This Choice: If Age >= 19 Age 19-25 (Q254_3)	○	○	○	○	○	○	○	○
Display This Choice: If Age >= 26 Age 26-29 (Q254_4)	○	○	○	○	○	○	○	○

Display This Choice: If Age >= 30 Age 30-39 (Q254_5)	○	○	○	○	○	○	○	○
Display This Choice: If Age >= 40 Age 40-49 (Q254_6)	○	○	○	○	○	○	○	○
Display This Choice: If Age >= 50 Age 50-59 (Q254_7)	○	○	○	○	○	○	○	○
Display This Choice: If Age >= 60 Age 60+ (Q254_8)	○	○	○	○	○	○	○	○

Page Break

Display This Question:

If Substance Use   The following questions are about your substance use/abuse. We are asking because... = Yes, and still do

*

Q192 **On average, how many cigarettes per day do you smoke now?**

____

Page Break

Display This Question:

If Substance Use The following questions are about your substance use/abuse. We are asking because... = Yes, but not currently

Q193 **At what age did you quit smoking cigarettes for the last time?**

Age: ____

Page Break

Q194 **For how many years total have you smoked cigarettes?**

Years:

____

Page Break

Q195 **Have you ever used any of the following products or any other tobacco-related products?**

- ☐ E-cigarettes (Juul, vaping) (1)
- ☐ Cigars (2)
- ☐ Chewing tobacco (3)
- ☐ Snus (4)
- ☐ Snuff (5)
- ☐ Hookah/shisha/water pipe (6)
- ☐ Betel nut/leaf (7)
- ☐ Clove cigarettes (Kretek) (8)
- ☐ Other: (9) ____
- ☐ ⊗None of the above (10)
- ☐ ⊗Prefer not to answer (999)

Q196 **Do you currently use any of the following products or any other tobacco-related products?**

- E-cigarettes (Juul, vaping) (1)
- Cigars (2)
- Chewing tobacco (3)
- Snus (4)
- Snuff (5)
- Hookah/shisha/water pipe (6)
- Betel nut/leaf (7)
- Clove cigarettes (Kretek) (8)
- Other: (9) ____
- ⊗None of the above (10)
- ⊗Prefer not to answer (999)

Page Break

Q197
An **alcoholic beverage** is defined as any of the following:

12 ounces of beer
8 ounces of malt liquor
5 ounces of wine
1.5 ounces or a "shot" of 80-proof distilled spirits or liquor (examples: gin, rum, vodka, whiskey)

Do you drink alcoholic beverages?

○ Yes (1)

○ No (0)

Page Break

Display This Question:

If An alcoholic beverage is defined as any of the following: 12 ounces of beer 8 ounces of malt li... = Yes

X→

Q198

An **alcoholic beverage** is defined as any of the following:

12 ounces of beer
8 ounces of malt liquor
5 ounces of wine
1.5 ounces or a "shot" of 80-proof distilled spirits or liquor (examples: gin, rum, vodka, whiskey)

Approximately how many alcoholic beverages did you consume <u>on a typical day that you drank any alcohol</u> during each age range listed below?

Display This Choice:

If Age >= 19

Display This Choice:

If Age >= 26

Display This Choice:

If Age >= 30

Display This Choice:

If Age >= 40

Display This Choice:

If Age >= 50

Display This Choice:

If Age >= 60

	0 (1)	1-3 (2)	4-5 (3)	6-9 (4)	10-14 (6)	15 or more (7)	Prefer not to answer (999)
Age (Q262_1)	○	○	○	○	○	○	○
Age 16-18 (Q262_2)	○	○	○	○	○	○	○
Display This Choice: If Age >= 19 Age 19-25	○	○	○	○	○	○	○

(Q262_3)							
Display This Choice: If Age >= 26 Age 26-29 (Q262_4)	○	○	○	○	○	○	○
Display This Choice: If Age >= 30 Age 30-39 (Q262_5)	○	○	○	○	○	○	○
Display This Choice: If Age >= 40 Age 40-49 (Q262_6)	○	○	○	○	○	○	○
Display This Choice: If Age >= 50 Age 50-59 (Q262_7)	○	○	○	○	○	○	○
Display This Choice: If Age >= 60 Age 60+ (Q262_8)	○	○	○	○	○	○	○

Display This Question:

If An alcoholic beverage is defined as any of the following: 12 ounces of beer 8 ounces of malt li... = Yes

Q199

An **alcoholic beverage** is defined as any of the following:

12 ounces of beer
8 ounces of malt liquor
5 ounces of wine
1.5 ounces or a "shot" of 80-proof distilled spirits or liquor (examples: gin, rum, vodka, whiskey)

Approximately how many <u>alcoholic beverages per week in a typical week</u> did you consume during each age range listed below?

Display This Choice:
If Age >= 19
Display This Choice:
If Age >= 26
Display This Choice:
If Age >= 30
Display This Choice:
If Age >= 40
Display This Choice:
If Age >= 50
Display This Choice:
If Age >= 60

	0 (1)	1-2 (2)	3-7 (3)	8-14 (4)	15-29 (6)	30 or more (7)	Prefer not to answer (999)
Age (Q262_1)	○	○	○	○	○	○	○
Age 16-18 (Q262_2)	○	○	○	○	○	○	○
Display This Choice: If Age >= 19 Age 19-	○	○	○	○	○	○	○

25 (Q262_3)							
Display This Choice: If Age >= 26 Age 26-29 (Q262_4)	○	○	○	○	○	○	○
Display This Choice: If Age >= 30 Age 30-39 (Q262_5)	○	○	○	○	○	○	○
Display This Choice: If Age >= 40 Age 40-49 (Q262_6)	○	○	○	○	○	○	○
Display This Choice: If Age >= 50 Age 50-59 (Q262_7)	○	○	○	○	○	○	○
Display This Choice: If Age >= 60 Age 60+ (Q262_8)	○	○	○	○	○	○	○

Page Break

Q200 **Have you ever used marijuana/cannabis/pot or hashish regularly?**

- ◯ Yes (1)
- ◯ No (0)

Skip To: Q207 If Have you ever used marijuana/cannabis/pot or hashish regularly? != Yes

Page Break

Q201 **At what age did you start using marijuana/cannabis/pot or hashish regularly?**

Age:

____

Q202 In a typical month, on how many days per month did you use **marijuana/cannabis/pot** or hashish during each age range listed below?

Display This Choice:

If Age >= 19

Display This Choice:

If Age >= 26

Display This Choice:

If Age >= 30

Display This Choice:

If Age >= 40

Display This Choice:

If Age >= 50

Display This Choice:

If Age >= 60

	None (1)	1 (2)	2-3 (3)	4-8 (4)	9-24 (6)	25-30 (7)	Prefer not to answer (999)	Don't know (888)
Age (Q265_1)	o	o	o	o	o	o	o	o
Age 16-18 (Q265_2)	o	o	o	o	o	o	o	o
Display This Choice: If Age >= 19 Age 19-25 (Q265_3)	o	o	o	o	o	o	o	o
Display This Choice: If Age >= 26 Age 26-29 (Q265_4)	o	o	o	o	o	o	o	o

Display This Choice: If Age >= 30 Age 30-39 (Q265_5)	○	○	○	○	○	○	○	○
Display This Choice: If Age >= 40 Age 40-49 (Q265_6)	○	○	○	○	○	○	○	○
Display This Choice: If Age >= 50 Age 50-59 (Q265_7)	○	○	○	○	○	○	○	○
Display This Choice: If Age >= 60 Age 60+ (Q265_8)	○	○	○	○	○	○	○	○

X→

Q203 **Do you currently use marijuana/cannabis/pot or hashish?**

○ Yes (1)

○ No (0)

Display This Question:

If Do you currently use marijuana/cannabis/pot or hashish? = Yes

X→

Q204 **How often do you currently use marijuana/cannabis/pot or hashish?**

○ Less than once per month (9)

○ Once per month (1)

○ 2-3 times per month (2)

○ 4-8 times per month (about 1-2 times per week) (3)

○ 9-24 times per month (about 3-6 times per week) (4)

○ 25-30 times per month (one or more times per day) (5)

○ Don't know (888)

○ Prefer not to answer (999)

Display This Question:

If Do you currently use marijuana/cannabis/pot or hashish? = Yes

X→

Q205 **What is the primary reason you use marijuana/cannabis/pot or hashish?**

○ Recreation (1)

○ Medical (2)

○ Other (3) ____

Page Break

Display This Question:

If Do you currently use marijuana/cannabis/pot or hashish? = No

Q206 **At what age did you stop using marijuana/cannabis/pot or hashish for the last time?**

Age:

____

Page Break

Q207 **Have you ever injected illegal drugs? If so, which ones?** (Do not include injection of drugs related to medical care or transitioning.)

- ☐ ⊗ I have never injected drugs (1)
- ☐ Steroids (2)
- ☐ Heroin (3)
- ☐ Cocaine (4)
- ☐ Crystal meth (5)
- ☐ Other: (6) ____
- ☐ ⊗ Prefer not to answer (999)

Page Break

Q208 **Have you ever snorted or smoked other drugs? If so, which ones?**

- ☐ ⊗ I have never snorted or smoked drugs (1)
- ☐ Steroids (2)
- ☐ Heroin (3)
- ☐ Cocaine (4)
- ☐ Crystal meth (5)
- ☐ Other: (6) ____
- ☐ ⊗ Prefer not to answer (999)

Page Break

Q209 **This next set of questions is about prescription pain medications, sometimes called opioids. Examples of these medications include pain relievers such as Vicodin, oxycodone, hydrocodone, Percocet, tramadol, and codeine-based products.**

Have you ever, even once, used any prescription pain reliever in any way a provider did not direct you to use it?

- Yes (1)
- No (0)

Display This Question:

If This next set of questions is about prescription pain medications, sometimes called opioids. Exam... = Yes

Q210 **In the past year, how many times did you use any prescription pain reliever in any way a medical provider did not direct you to use it?**

- 0 times (1)
- 1 or 2 times (2)
- 3 to 9 times (3)
- More than 10 times (4)
- Every day (5)

Display This Question:

If This next set of questions is about prescription pain medications, sometimes called opioids. Exam... = Yes

Q211 **If have you obtained prescription pain reliever that you used in any way a provider did not direct you to use it, how did you get it? Please check all that apply**

- ☐ I've used a friend/partner's prescription pain reliever with their knowledge (1)
- ☐ I've used a friend/partner's prescription pain reliever without their knowledge (2)
- ☐ I've bought a prescription pain reliever off the internet (3)
- ☐ I've used a prescription pain reliever that was originally prescribed for me by a provider (4)
- ☐ I obtained my prescription pain reliever another way (please specify) (5) ____

End of Block: Substance Use
