# Supplementary material for: Prevalence and Determinants of Cervicovaginal, Oral, and Anal Human Papillomavirus Infection in a Population of Transgender and Gender Diverse People Assigned Female at Birth
Source: LGBT Health. 2024 Sep 5;11(6):437–45. doi: 10.1089/lgbt.2023.0335 (PMC11449398; doi:10.1089/lgbt.2023.0335)
Supplement: Supplementary Table S1 [file lgbt.2023.0335_suppl_tables1.pdf]

Supplementary Table S1: Comparison participants who completed at least one study product vs those who completed the sociodemographic and behavioral questionnaire and returned their biospecimens.

	Completed questionnaire and biospecimens (N=102)		Completed questionnaire (N=112)	
	n	%	n	%
Age Group				
21-29	67	65.7%	73	65.1%
30-39	29	28.4%	29	25.9%
40-59	6	5.9%	10	8.9%
Race/Ethnicity				
White	84	82.4%	93	83.0%
Multiracial	8	7.8%	9	8.0%
Asian	5	4.9%	5	4.5%
Black	5	4.9%	5	4.5%
Gender				
Nonbinary, genderfluid, agender	49	48.0%	57	50.9%
Male, transgender male, transmasculine	50	49.0%	52	46.4%
Other / Prefer not to label	3	2.9%	3	2.7%
Sexual Orientation				
Bisexual, pansexual, or omnisexual	44	43.1%	47	42.0%
Queer	37	36.3%	41	36.6%
Homosexual	9	8.8%	12	10.7%
Asexual or demisexual	8	7.8%	8	7.1%
Straight or heterosexual	3	2.9%	3	2.7%
Other / Prefer not to label	1	1.0%	1	0.9%
Highest Level of Education				
High school or less	8	7.8%	9	8.0%
Some college	27	26.5%	30	26.8%

2- or 4- year degree	46	45.1%	51	45.5%
Professional or doctorate degree	21	20.6%	22	19.6%
<u>Current Employment Status</u>				
Full time	38	37.3%	40	35.7%
Student	22	21.6%	24	21.4%
Part time	15	14.7%	17	15.2%
Unemployed, looking for work	12	11.8%	14	12.5%
Disabled and not working	10	9.8%	10	8.9%
Unemployed, not looking for work	5	4.9%	6	5.4%
<u>Yearly Household Income</u>				
Less than $10,000	17	16.8%	17	15.2%
$10,000 - $19,999	19	18.8%	20	17.9%
$20,000 - $39,999	20	19.8%	23	20.5%
$40,000 - $49,999	21	20.8%	22	19.6%
$50,000 +	24	23.8%	29	26.9%
<u>Developed Environment</u>				
Suburban	49	48.0%	55	49.1%
Urban	47	46.1%	50	44.6%
Rural	6	5.9%	7	6.3%
<u>Marital Status</u>				
Single partner, married	47	46.1%	51	45.5%
Never married or partnered	25	24.5%	26	23.2%
Single partner, never married	12	11.8%	14	12.5%
Multiple committed partners	11	10.8%	12	10.7%
Separated, divorced, or widowed	7	6.9%	9	8.0%

N is the number of participants answering the question, and n is the number of respondents answering in the affirmative.
