# Supplementary material for: Prevalence and Determinants of Cervicovaginal, Oral, and Anal Human Papillomavirus Infection in a Population of Transgender and Gender Diverse People Assigned Female at Birth
Source: LGBT Health. 2024 Sep 5;11(6):437–45. doi: 10.1089/lgbt.2023.0335 (PMC11449398; doi:10.1089/lgbt.2023.0335)
Supplement: Supplementary Table S2 [file lgbt.2023.0335_suppl_tables2.pdf]

Supplementary Table S2. Prevalence of human papillomavirus genotypes in each sample type.

HPV Genotype	Oral (91 Valid Tests)		Cervicovaginal (82 Valid Tests)		Anal (48 Valid Tests)	
	Number Positive	%	Number Positive	%	Number Positive	%
Any	8	8.8%	25	30.5%	19	39.6%
HPV 6	0	0.0%	2	2.4%	3	6.3%
HPV 11	0	0.0%	0	0.0%	0	0.0%
HPV 16	1	1.1%	3	3.7%	3	6.3%
HPV 18	2	2.2%	5	6.1%	1	2.1%
HPV 31	0	0.0%	0	0.0%	0	0.0%
HPV 33	0	0.0%	0	0.0%	1	2.1%
HPV 35	0	0.0%	0	0.0%	2	4.2%
HPV 39	1	1.1%	3	3.7%	3	6.3%
HPV 45	0	0.0%	1	1.2%	1	2.1%
HPV 51	1	1.1%	6	7.3%	4	8.3%
HPV 52	0	0.0%	4	4.9%	1	2.1%
HPV 56	1	1.1%	5	6.1%	4	8.3%
HPV 58	0	0.0%	1	1.2%	0	0.0%
HPV 59	0	0.0%	5	6.1%	2	4.2%
HPV 66	2	2.2%	3	3.7%	3	6.3%
HPV 68	0	0.0%	3	3.7%	0	0.0%
HPV 73	0	0.0%	2	2.4%	0	0.0%
HPV 90	0	0.0%	1	1.2%	1	2.1%
cobas test genotypes (HPV 16, 18, 31, 33, 35, 39, 45, 51, 52, 56, 58, 59, 66, and 68)	8	8.8%	23	28.1%	18	37.5%
High-risk genotypes (IARC Group 1) (HPV 16, 18, 31, 33, 35, 39, 45, 51, 52, 56, 58, and 59)	6	6.6%	22	26.8%	16	33.3%
Genotypes included in Gardasil 9 (HPV 6, 11, 16, 18, 31, 33, 45, 52, and 58)	3	3.3%	14	17.1%	8	16.7%
Genotypes included in original Gardasil (HPV 6, 11, 16, and 18)	3	3.3%	10	12.2%	7	14.6%
Genotypes causing the majority of cervical cancer (HPV 16 and 18)	3	3.3%	8	9.7%	4	8.3%
Genotypes causing genital warts (HPV 6 and 11)	0	0.0%	2	2.4%	3	6.3%

Abbreviations: HPV (human papillomavirus), IARC (International Agency on Research on Cancer)
