# Supplementary material for: Prevalence and Determinants of Cervicovaginal, Oral, and Anal Human Papillomavirus Infection in a Population of Transgender and Gender Diverse People Assigned Female at Birth
Source: LGBT Health. 2024 Sep 5;11(6):437–45. doi: 10.1089/lgbt.2023.0335 (PMC11449398; doi:10.1089/lgbt.2023.0335)
Supplement: Supplementary Table S3 [file lgbt.2023.0335_suppl_tables3.pdf]

**Supplementary Table S3.** Genotype concordance between anal and cervicovaginal and between oral and cervicovaginal human papillomavirus infections.

| | Anal/cervicovaginal genotype matches (44 participants with valid tests for both samples) | | | Oral/cervicovaginal genotype matches (75 participants with valid tests for both samples) | | |
|---|---|---|---|---|---|---|
| HPV Genotype | Number positive for both | % of positive cervicovaginal tests | % of positive anal tests | Number positive for both | % of positive cervicovaginal tests | % of positive oral tests |
| Any | 9[a] | 56.3% | 50.0% | 5 | 20.8% | 71.4% |
| HPV 6 | 2 | 100.0% | 66.7% | 0 | 0.0% | - |
| HPV 11 | 0 | - | - | 0 | - | - |
| HPV 16 | 1 | 33.3% | 33.3% | 0 | 0.0% | 0.0% |
| HPV 18 | 0 | 0.0% | 0.0% | 1 | 25.0% | 50.0% |
| HPV 31 | 0 | - | - | 0 | - | - |
| HPV 33 | 0 | - | - | 0 | - | - |
| HPV 35 | 0 | - | - | 0 | - | - |
| HPV 39 | 2 | 100.0% | 66.7% | 0 | - | - |
| HPV 45 | 0 | - | - | 0 | - | - |
| HPV 51 | 1 | 33.3% | 25.0% | 1 | 16.7% | 100.0% |
| HPV 52 | 1 | 25.0% | 100.0% | 0 | - | - |
| HPV 56 | 2 | 50.0% | 50.0% | 1 | 20.0% | 100.0% |
| HPV 58 | 0 | - | - | 0 | - | - |
| HPV 59 | 2 | 50.0% | 100.0% | 0 | - | - |
| HPV 66 | 1 | 100.0% | 50.0% | 2 | 66.7% | 100.0% |
| HPV 68 | 0 | - | - | 0 | - | - |
| HPV 73 | 0 | - | - | 0 | - | - |
| HPV 90 | 1 | 100.0% | 100.0% | 0 | - | - |

Abbreviations: HPV (human papillomavirus)

a: Individuals could be concordant for multiple genotypes
