# Supplementary material for: Prevalence and Determinants of Cervicovaginal, Oral, and Anal Human Papillomavirus Infection in a Population of Transgender and Gender Diverse People Assigned Female at Birth
Source: LGBT Health. 2024 Sep 5;11(6):437–45. doi: 10.1089/lgbt.2023.0335 (PMC11449398; doi:10.1089/lgbt.2023.0335)
Supplement: Supplementary Table S4 [file lgbt.2023.0335_suppl_tables4.pdf]

Supplementary Table S4: Human papillomavirus prevalence and prevalence ratios by site and participant demographic, behavioral, and medical characteristics.

	Oral HPV (91 Valid Tests)				Cervicovaginal HPV (82 Valid Tests)				Anal HPV (48 Valid Tests)			
	N	P	PR	95% CI	N	P	PR	95% CI	N	P	PR	95% CI
Total	91	8.8%	-	-	82	30.5%	-	-	48	39.6%	-	-
Age Group	91	-	-	-	82	-	-	-	48	-	-	-
21-29	61	11.5%	1.00	(Ref)	58	32.8%	1.00	(Ref)	34	41.2%	1.00	(Ref)
30-39	25	4.0%	0.35	0.05, 2.69	21	19.1%	0.58	0.22, 1.51	14	35.7%	0.87	0.39, 1.95
40-59	5	0.0%	0.00	-	3	†	†	†	0	-	-	-
Race/Ethnicity	91	-	-	-	82	-	-	-	48	-	-	-
White	73	6.9%	1.00	(Ref)	67	26.9%	1.00	(Ref)	39	38.5%	1.00	(Ref)
Multiracial	8	25.0%	3.65	0.84, 15.9	7	28.6%	1.06	0.31, 3.66	3	†	†	†
Asian	5	0.0%	0.00	-	4	†	†	†	3	†	†	†
Black	5	20.0%	2.92	0.42, 20.5	4	†	†	†	3	†	†	†
Gender	91	-	-	-	82	-	-	-	48	-	-	-
Non-binary, genderfluid, agender	44	4.6%	1.00	(Ref)	37	18.9%	1.00	(Ref)	23	30.4%	1.00	(Ref)
Male, transgender male, transmasculine	44	11.4%	2.50	0.51, 12.2	42	38.1%	2.01	0.93, 4.35	23	47.8%	1.57	0.74, 3.33
Sexual Attraction	91	-	-	-	82	-	-	-	48	-	-	-
Bisexual, pansexual, omnisexual	41	7.8%	1.00	(Ref)	39	33.3%	1.00	(Ref)	21	42.9%	1.00	(Ref)
Queer	31	6.5%	0.66	0.13, 3.38	28	14.3%	0.43	0.16, 1.18	15	26.7%	0.62	0.24, 1.65
Homosexual	9	22.2%	2.28	0.49, 10.59	7	71.4%	2.14	1.12, 4.09	6	83.3%	**1.94**	**1.06, 3.58**
Asexual or demisexual	6	0.0%	0.00	-	4	†	†	†	3	†	†	†
Straight or heterosexual	3	†	†	†	3	†	†	†	2	†	†	†
Highest Level of Education	91	-	-	-	82	-	-	-	48	-	-	-
High school or less	8	0.0%	0.00	-	5	60.0%	1.63	0.71, 3.73	5	60.0%	1.58	0.64, 3.87
Some college	24	12.5%	1.31	0.32, 5.38	23	21.7%	0.59	0.24, 1.42	13	38.5%	1.01	0.42, 2.43
2- or 4- year degree	42	9.5%	1.00	(Ref)	38	36.8%	1.00	(Ref)	21	38.1%	1.00	(Ref)

Professional/doctorate degree	17	5.9%	0.62	0.07, 5.13	16	18.8%	0.51	0.17, 1.53	9	33.3%	0.88	0.30, 2.56
<u>Current Employment Status</u>	91	-	-	-	82	-	-	-	48	-	-	-
Full time	35	17.1%	1.00	(Ref)	30	43.3%	1.00	(Ref)	21	42.9%	1.00	(Ref)
Student	19	10.5%	0.61	0.14, 2.75	18	27.8%	0.64	0.27, 1.50	10	30.0%	0.70	0.24, 2.04
Part time	14	0.0%	0.00	-	11	9.1%	0.21	0.03, 1.42	6	33.3%	0.78	0.23, 2.67
Unemployed, looking for work	9	0.0%	0.00	-	9	33.3%	0.77	0.28, 2.11	4	†	†	†
Disabled and not working	10	0.0%	0.00	-	9	22.2%	0.51	0.14, 1.86	5	40.0%	0.93	0.29, 3.04
Unemployed, not looking	4	†	†	†	5	20.0%	0.46	0.08, 2.79	2	†	†	†
<u>Yearly Household Income</u>	90	-	-	-	81	-	-	-	47	-	-	-
Less than $10,000	16	6.3%	1.00	(Ref)	13	30.8%	1.00	(Ref)	5	60.0%	1.00	(Ref)
$10,000 - $19,999	17	5.9%	0.94	0.06, 13.8	16	37.5%	1.22	0.43, 3.42	12	50.0%	0.83	0.33, 2.08
$20,000 - $39,999	16	12.5%	2.00	0.20, 19.9	18	50.0%	1.63	0.64, 4.15	11	36.4%	0.61	0.21, 1.75
$40,000 - $49,999	18	22.2%	3.56	0.44, 28.6	17	17.7%	0.57	0.15, 2.13	11	25.0%	0.61	0.21, 1.75
$50,000 +	23	0.0%	0.00	-	17	17.7%	0.57	0.15, 2.13	8	40.4%	0.42	0.10, 1.69
<u>Developed Environment</u>	91	-	-	-	82	-	-	-	48	-	-	-
Suburban	44	9.1%	1.00	(Ref)	35	28.6%	1.00	(Ref)	18	38.9%	1.00	(Ref)
Urban	41	7.3%	0.80	0.19, 3.38	41	29.3%	1.02	0.50, 2.08	28	39.3%	1.01	0.48, 2.12
Rural	6	16.7%	1.83	0.24, 13.8	6	50.0%	1.75	0.67, 4.55	2	50.0%	1.29	0.29, 5.77
<u>Marital Status</u>	91	-	-	-	82	-	-	-	48	-	-	-
Single partner, married	43	14.0%	1.00	(Ref)	39	25.6%	1.00	(Ref)	21	38.1%	1.00	(Ref)
Never married or partnered	25	8.0%	0.57	0.13, 2.63	22	45.5%	1.77	0.88, 3.58	16	43.8%	1.15	0.53, 2.50
Single partner, never married	10	0.0%	0.00	-	8	12.5%	0.49	0.07, 3.29	3	†	†	†
Multiple committed partners	7	0.0%	0.00	-	7	28.6%	1.11	0.31, 4.04	6	33.3%	0.88	0.25, 3.07
Separated/divorced/widowed	6	0.0%	0.00	-	6	33.3%	1.30	0.37, 4.54	2	†	†	†
<u>Partners with a Vagina, Deep Kissing, Last 6 Months</u>	90	-	-	-	80	-	-	-	48	-	-	-
Never	3	†	†	†	1	†	†	†	0	†	†	†
0	40	5.0%	1.00	(Ref)	38	36.8%	1.00	(Ref)	24	50.0%	1.00	(Ref)
1	39	12.8%	2.56	0.53, 12.44	35	20.0%	0.54	0.25, 1.19	18	22.2%	0.44	0.17, 1.15

2 - 4	8	12.5%	2.50	0.26, 24.38	6	66.7%	1.81	0.90, 3.65	6	50.0%	1.00	0.41, 2.45
Partners with a Penis, Deep Kissing, Last 6 Months	90	-	-	-	80	-	-	-	48	-	-	-
Never	5	0.00%	0.00	-	3	†	†	†	1	†	†	†
0	42	11.9%	1.00	(Ref)	39	25.6%	1.00	(Ref)	15	13.3%	1.00	(Ref)
1	32	9.4%	0.79	0.20, 3.05	28	35.7%	1.39	0.67, 2.89	22	50.0%	3.75	0.97, 14.6
2 - 4	5	0.0%	0.00	-	5	20.0%	0.78	0.12, 4.88	5	40.0%	3.00	0.56, 16.1
5 +	6	0.0%	0.00	-	5	80.0%	**3.12**	**1.56, 6.23**	5	80.0%	**6.00**	**1.54, 23.4**
Partners with a Vagina, Any Sex, Last 6 Months	91	-	-	-	82	-	-	-	48	-	-	-
Never	5	0.0%	0.00	-	3	†	†	†	1	†	†	†
0	48	6.3%	1.00	(Ref)	45	35.6%	1.00	(Ref)	30	46.7%	1.00	(Ref)
1	33	12.1%	1.94	0.46, 8.10	30	20.0%	0.56	0.25, 1.27	13	23.1%	0.49	0.17, 1.43
2 - 4	5	0.0%	0.00	-	4	†	†	†	4	†	†	†
Partners with a Penis, Any Sex, Last 6 Months	91	-	-	-	82	-	-	-	48	-	-	-
Never	5	0.0%	0.00	-	3	0.0%	0.00	-	1	0.0%	0.00	-
0	46	10.9%	1.00	(Ref)	44	27.3%	1.00	(Ref)	17	17.7%	1.00	(Ref)
1	31	9.7%	0.89	0.23, 3.46	27	33.3%	1.22	0.59, 2.51	22	50.0%	2.83	0.94, 8.6
2 - 4	6	0.0%	0.00	-	5	20.0%	0.73	0.12, 4.52	5	40.0%	2.27	0.51, 10.0
5 +	3	†	†	†	3	†	†	†	3	†	†	†
Partners with a Vagina, Vaginal/Front Hole Sex, Last 6 Months	89	-	-	-	81	-	-	-	46	-	-	-
Never	5	0.0%	0.00	-	3	0.0%	0.00	-	1	0.0%	0.00	-
0	43	4.7%	1.00	(Ref)	41	34.2%	1.00	(Ref)	27	48.2%	1.00	(Ref)
1	37	13.5%	2.91	0.60, 14.10	33	21.2%	0.62	0.28, 1.36	15	20.0%	0.42	0.14, 1.23
2 - 4	4	†	†	†	4	†	†	†	3	†	†	†
Partners with a Penis, Vaginal/Front Hole Sex, Last 6 Months	89	-	-	-	81	-	-	-	46	-	-	-
Never	5	0.0%	0.00	-	3	0.0%	0.00	-	1	0.0%	0.00	-
0	47	8.5%	1.00	(Ref)	45	24.4%	1.00	(Ref)	18	16.7%	1.00	(Ref)
1	26	11.5%	1.36	0.33, 5.60	23	39.1%	1.60	0.78, 3.30	18	44.4%	2.67	0.84, 8.46

2 - 4	8	0.0%	0.00	-	7	14.3%	0.58	0.09, 3.85	6	50.0%	3.00	0.81, 11.1
5 +	3	†	†	†	3	†	†	†	3	†	†	†
Partners with a Vagina, Participant Receiving Oral Sex, Last 6 Months	48	-	-	-	44	-	-	-	23	-	-	-
Never	5	0.0%	0.00	-	3	†	†	†	1	†	†	†
0	11	9.1%	0.59	0.07, 4.71	10	0.0%	0.00	-	6	16.7%	0.92	0.10, 8.15
1	26	15.4%	1.00	(Ref)	26	23.1%	1.00	(Ref)	11	18.2%	1.00	(Ref)
2 - 4	6	16.7%	1.08	0.15, 8.03	5	80.0%	**3.47**	**1.52, 7.93**	5	60.0%	3.30	0.78, 20.0
Partners with a Penis, Participant Receiving Oral Sex, Last 6 Months	41	-	-	-	36	-	-	-	29	-	-	-
Never	5	0.0%	0.00	-	3	†	†	†	1	†	†	†
0	3	†	†	†	3	†	†	†	1	†	†	†
1	25	8.0%	1.00	(Ref)	22	27.3%	1.00	(Ref)	19	42.1%	1.00	(Ref)
2 - 4	5	0.0%	0.00	-	5	20.0%	0.73	0.11, 4.81	5	60.0%	1.43	0.59, 3.47
5 +	3	†	†	†	3	†	†	†	3	†	†	†
Partners with a Vagina, Participant Performing Oral Sex, Last 6 Months	48	-	-	-	44	-	-	-	23	-	-	-
Never	5	0.0%	0.00	-	3	†	†	†	1	†	†	†
0	8	0.0%	0.00	-	8	0.0%	0.00	-	4	†	†	†
1	30	16.7%	1.00	(Ref)	29	24.1%	1.00	(Ref)	14	28.6%	1.00	(Ref)
2 - 4	5	20.0%	1.20	0.17, 8.24	4	†	†	†	4	†	†	†
Partners with a Penis, Participant Performing Oral Sex, Last 6 Months	41	-	-	-	36	-	-	-	29	-	-	-
Never	5	0.0%	0.00	-	3	†	†	†	1	†	†	†
0	1	†	†	†	0	†	†	†	0	†	†	†
1	27	11.1%	1.00	(Ref)	25	36.0%	1.00	(Ref)	20	45.0%	1.00	(Ref)
2 - 4	5	0.0%	0.00	-	5	20.0%	0.56	0.09, 3.46	5	60.0%	1.33	0.56, 3.16
5 +	3	†	†	†	3	†	†	†	3	†	†	†
Partners with a Vagina, Anal Sex, Last 6 Months	56	-	-	-	51	-	-	-	32	-	-	-

Never	5	0.0%	0.00	-	3	†	†	†	1	†	†	†
0	39	7.7%	1.00	(Ref)	36	30.6%	1.00	(Ref)	27	40.7%	1.00	(Ref)
1	12	8.3%	1.08	0.12, 9.48	12	33.3%	1.09	0.43, 2.79	4	25.0%	0.61	0.11, 3.56
Partners with a Penis, Anal Sex, Last 6 Months	56	-	-	-	51	-	-	-	32	-	-	-
Never	5	0.0%	0.00	-	3	†	†	†	1	†	†	†
0	35	5.7%	1.00	(Ref)	35	28.6%	1.00	(Ref)	20	30.0%	1.00	(Ref)
1	14	14.3%	2.50	0.39, 16.05	11	45.5%	1.59	0.69, 3.66	9	55.6%	1.85	0.76, 4.50
2 - 4	2	†	†	†	2	†	†	†	2	†	†	†
Transitioned/Transitioning	91	-	-	-	82	-	-	-	48	-	-	-
Yes	69	7.3%	1.00	(Ref)	66	31.8%	1.00	(Ref)	36	41.7%	1.00	(Ref)
No	22	13.6%	1.88	0.49, 7.25	16	25.0%	0.79	0.31, 1.97	12	33.3%	0.80	0.33, 1.95
Ever Taken Hormones for Gender-Affirming Treatment	68	-	-	-	65	-	-	-	36	-	-	-
Yes	50	8.0%	1.00	(Ref)	51	33.3%	1.00	(Ref)	29	44.8%	1.00	(Ref)
No	18	5.6%	0.69	0.08, 5.81	14	21.4%	0.64	0.22, 1.88	7	28.6%	0.64	0.18, 2.20
Ever Had Gender-Affirming Surgery	69	-	-	-	66	-	-	-	36	-	-	-
Yes	28	7.1%	0.98	0.17, 5.47	28	35.7%	1.23	0.61, 2.49	16	50.0%	1.43	0.66, 3.09
No	41	7.3%	1.00	(Ref)	38	29.0%	1.00	(Ref)	20	35.0%	1.00	(Ref)
Ever Been Pregnant	91				82				48			
Yes	13	0.0%	0.00	-	9	33.3%	1.11	0.41, 2.97	6	16.7%	0.39	0.06, 2.41
No	78	10.3%	1.00	(Ref)	73	30.1%	1.00	(Ref)	42	42.9%	1.00	(Ref)
Alcohol Use	89	-	-	-	80	-	-	-	47	-	-	-
Current	66	10.6%	1.00	(Ref)	61	27.9%	1.00	(Ref)	35	37.1%	1.00	(Ref)
Non-current	23	4.4%	0.41	0.05, 3.15	19	36.8%	1.32	0.65, 2.70	12	41.7%	1.12	0.51, 2.49
Marijuana Use	89	-	-	-	80	-	-	-	47	-	-	-
Ever	46	17.4%	1.00	(Ref)	48	33.3%	1.00	(Ref)	28	46.4%	1.00	(Ref)
Never	43	0.0%	0.00	-	32	25.0%	0.75	0.36, 1.54	19	26.3%	0.57	0.24, 1.33
Smoking Status	89	-	-	-	80	-	-	-	47	-	-	-
Never	62	9.7%	1.00	(Ref)	56	28.6%	1.00	(Ref)	31	38.7%	1.00	(Ref)
Previous	21	9.5%	0.98	0.21, 4.51	20	25.0%	0.88	0.37, 2.08	12	25.0%	0.65	0.22, 1.89
Current	6	0.0%	0.00	-	4	†	†	†	4	†	†	†
HPV Vaccination	91	-	-	-	82	-	-	-	48	-	-	-

At least one dose	53	9.4%	1.00	(Ref)	52	30.8%	1.00	(Ref)	27	29.6%	1.00	(Ref)
None	38	7.9%	0.84	0.21, 3.29	30	30.0%	0.98	0.49, 1.93	21	52.4%	1.77	0.87, 3.60
Ever Had a Cervical Pap Smear	91	-	-	-	82	-	-	-	48	-	-	-
Yes	74	9.5%	1.00	(Ref)	66	31.8%	1.00	(Ref)	39	43.6%	1.00	(Ref)
No	17	5.9%	0.62	0.08, 4.72	16	25.0%	0.79	0.31, 1.97	7	22.2%	0.51	0.14, 1.82
Ever Had an Anal Pap Smear	91	-	-	-	82	-	-	-	48	-	-	-
Yes	6	16.7%	2.02	0.30, 13.87	4	†	†	†	4	†	†	†
No	85	8.2%	1.00	(Ref)	78	28.2%	1.00	(Ref)	44	34.1%	1.00	(Ref)
History of HPV	91	-	-	-	82	-	-	-	48	-	-	-
Yes	8	0.0%	0.00	-	7	71.4%	**2.68**	**1.47, 4.88**	5	60.0%	1.61	0.71, 3.64
No	83	9.6%	1.00	(Ref)	75	26.7%	1.00	(Ref)	43	37.2%	1.00	(Ref)
History of other STI	89	-	-	-	82	-	-	-	47	-	-	-
Yes	20	5.0%	0.58	0.07, 4.50	20	50.0%	**2.07**	**1.11, 3.85**	15	66.7%	**2.67**	**1.33, 5.36**
No	69	8.7%	1.00	(Ref)	62	24.2%	1.00	(Ref)	32	25.0%	1.00	(Ref)
Medical Insurance	89	-	-	-	80	-	-	-	48	-	-	-
Private insurance	39	10.3%	1.00	(Ref)	33	30.3%	1.00	(Ref)	19	36.8%	1.00	(Ref)
Public insurance	28	7.1%	0.70	0.14, 3.54	27	33.3%	1.10	0.52, 2.31	18	55.6%	1.51	0.73, 3.10
Parents' private insurance	22	9.1%	0.89	0.18, 4.46	20	30.0%	0.99	0.42, 2.31	11	18.2%	0.49	0.12, 1.97
Ever Delayed Checkups	91	-	-	-	82	-	-	-	48	-	-	-
Yes, to avoid dysphoria or physical discomfort	53	7.6%	1.00	(Ref)	51	29.4%	1.00	(Ref)	28	53.6%	1.00	(Ref)
Yes, fear of discrimination	15	6.7%	0.88	0.11, 7.32	12	41.7%	1.42	0.64, 3.13	6	0.0%	0.00	-
Yes, provider lacked knowledge of trans-specific care	5	0.0%	0.00	-	5	20.0%	0.68	0.11, 4.13	4	†	†	†
No	18	16.7%	2.21	0.55, 8.94	14	28.6%	0.97	0.38, 2.47	10	30.0%	0.56	0.20, 1.53
Ever Delayed Treatment for Injury or Illness	91	-	-	-	82	-	-	-	48	-	-	-
Yes, to avoid dysphoria or physical discomfort	32	3.1%	0.23	0.03, 1.81	30	33.3%	1.30	0.62, 2.71	16	56.3%	1.85	0.87, 3.93
Yes, fear of discrimination	12	8.3%	0.61	0.08, 4.60	10	50.0%	1.95	0.86, 4.42	8	25.0%	0.82	0.21, 3.93

Yes, provider lacked knowledge of trans-specific care	3	†	†	†	3	†	†	†	1	†	†	†
No	44	13.6%	1.00	(Ref)	39	25.6%	1.00	(Ref)	23	30.4%	1.00	(Ref)

Significantly different prevalence ratios are highlighted and bolded. Bolded values are statistically significant at level of significance α=0.05.

N = number of individuals with valid test results in each group, P = prevalence of HPV within each group, PR = prevalence ratio of HPV in each group compared to referent group, and 95% CI = 95% confidence interval for each prevalence ratio.

Abbreviations: HPV (human papillomavirus), STI (sexually transmitted infection).

†Cells with fewer than 5 participants are censored.
